# Divergent Cytokine and Chemokine Responses at Early Acute Simian Immunodeficiency Virus Infection Correlated with Virus Replication and CD4 T Cell Loss in a Rhesus Macaque Model

**DOI:** 10.3390/vaccines11020264

**Published:** 2023-01-25

**Authors:** Nongthombam Boby, Apurv Srivastav, Sudesh K. Srivastav, Bapi Pahar

**Affiliations:** 1 Division of Comparative Pathology, Tulane National Primate Research Center, Covington, LA 70433, USA; 2 Department of Public Health Sciences, School of Medicine, University of California, Davis, CA 95816, USA; 3 Department of Biostatistics and Data Science, Tulane University, New Orleans, LA 70118, USA; 4 School of Medicine, Tulane University, New Orleans, LA 70118, USA

**Keywords:** acute infection, cytokines, chemokines, peripheral blood, rhesus macaque, SIV

## Abstract

Cytokine and chemokine levels remain one of the significant predictive factors of HIV pathogenesis and disease outcome. Understanding the impact of cytokines and chemokines during early acute infection will help to recognize critical changes during HIV pathogenesis and might assist in establishing improved HIV treatment and prevention methods. Sixty-one cytokines and chemokines were evaluated in the plasma of an SIV-infected rhesus macaque model. A substantial change in 11 cytokines/growth factors and 9 chemokines were observed during acute infection. Almost all the cytokines/chemokines were below the baseline values for an initial couple of days of infection. We detected six important cytokines/chemokines, such as IL-18, IP-10, FLT3L, MCP-1, MCP-2, and MIP-3β, that can be used as biomarkers to predict the peripheral CD4+ T cell loss and increased viral replication during the acute SIV/HIV infection. Hence, regulating IL-18, IP-10, FLT3L, MCP-1, MCP-2, and MIP-3β expression might provide an antiviral response to combat acute SIV/HIV infection.

## 1. Introduction

HIV, the causative agent of AIDS, is one of the world’s most serious public health-related sexually transmissible diseases, with 38.4 million people living with HIV infection as of 2021 (UNAIDS 2022 epidemiological estimates; aidsinfo.unaids.org). However, until today, more than 10 million HIV-infected people have no access to antiretroviral therapy (ART) (aidsinfo.unaids.org) despite its first reported case in June of 1981 [1]. ART has reduced AIDS mortality significantly. However, the treatment is not curative and the latent virus rebounds if the ART treatment is discontinued. Cytokine dysregulation and chronic inflammation are major clinical manifestations of HIV or simian immunodeficiency virus (SIV) infection [2,3,4,5,6,7]. HIV/SIV causes the early depletion of CD4+ T cells and induces dysregulation of protective immune responses, including the lack of antiviral mechanism and delay in the generation of effective neutralizing antibodies. The early depletion of intestinal CD4+ T cells during HIV/SIV infection was also associated with changes in both T-helper 1 (T_h_1) and T_h_2 cytokines that were also indicative of the failure of functional adaptive immune responses and the dysregulation of protective immunity [3,4]. In our earlier studies, a lack of T_h_1 to T_h_2 shift, as well as T cytotoxic 1(T_C_1) to T_C_2 cytokine shift in CD4+ and CD8+ T cells, respectively, in cells isolated from the intestine, peripheral blood, bone marrow, and axillary lymph node (LN) at 21 days post-SIV infection were detected when compared to the pre-infection phase [3,4]. However, limited data are available on the dynamics of different cytokines, chemokines, and growth factors at the early acute period of HIV infection and how those are related to the viral dynamics and peripheral CD4+ T cell loss. Since the cytokine and chemokine statuses remain one of the significant predictive factors of the viral load, disease progression, and the depletion of CD4+ T cells, understanding the significance of cytokines and chemokines during early acute infection will help to recognize critical changes during HIV pathogenesis. The knowledge will also help manage infected individuals’ clinical outcomes and assist in establishing improved HIV treatment and prevention methods.

In the present study, we have assessed 61 different cytokines and chemokines in plasma at the early acute SIV infection starting from 0, 0.25 (6 h.), 1, 2, 3, 4, 5, 7, 14, and 21 days of an SIV-infected rhesus macaque (RhM) model. RhMs remain an invaluable animal model for understanding HIV pathogenesis, which will mimic the cytokine/chemokine changes that happen during acute HIV infection in humans. Study related to early acute HIV infection in humans is likely impossible to implement. This study will provide insight into the cytokine status at the early stage of disease and has the potential to identify novel biomarkers that can predict virus replication and peripheral CD4+ T cell levels during early infection.

## 2. Materials and Methods

### 2.1. Ethics Statement

All Indian Rhesus macaques (RhMs, *Macaca mulatta*) were housed at the Tulane National Primate Research Center (TNPRC) biosafety level 2 facility by the standards incorporated in the guide for the cure and use of laboratory animals. The study was reviewed and approved by Tulane University IACUC protocol number 3818. TNPRC is fully accredited by AALAC, Animal Welfare Assurance No. A4499-01.

Macaques were singly housed indoors in climate-controlled buildings with a 12/12-light/dark cycle and were fed a commercially prepared nonhuman primate diet twice daily, supplemented by different feeding enrichments. Water was accessible ad libitum in each cage. All the subjects were monitored twice daily for pain, distress, and disease signs. In addition, the subjects were anesthetized intramuscularly with ketamine hydrochloride (10 mg/kg of BW) or tiletamine hydrochloride/zolazepam (Telazol, Zoetis, Parsippany, NJ, USA) (5–8 mg/kg of BW) for blood collection, physical exams, and virus inoculation [8].

### 2.2. SIV Infection and Sample Collection

**Figure 1:**
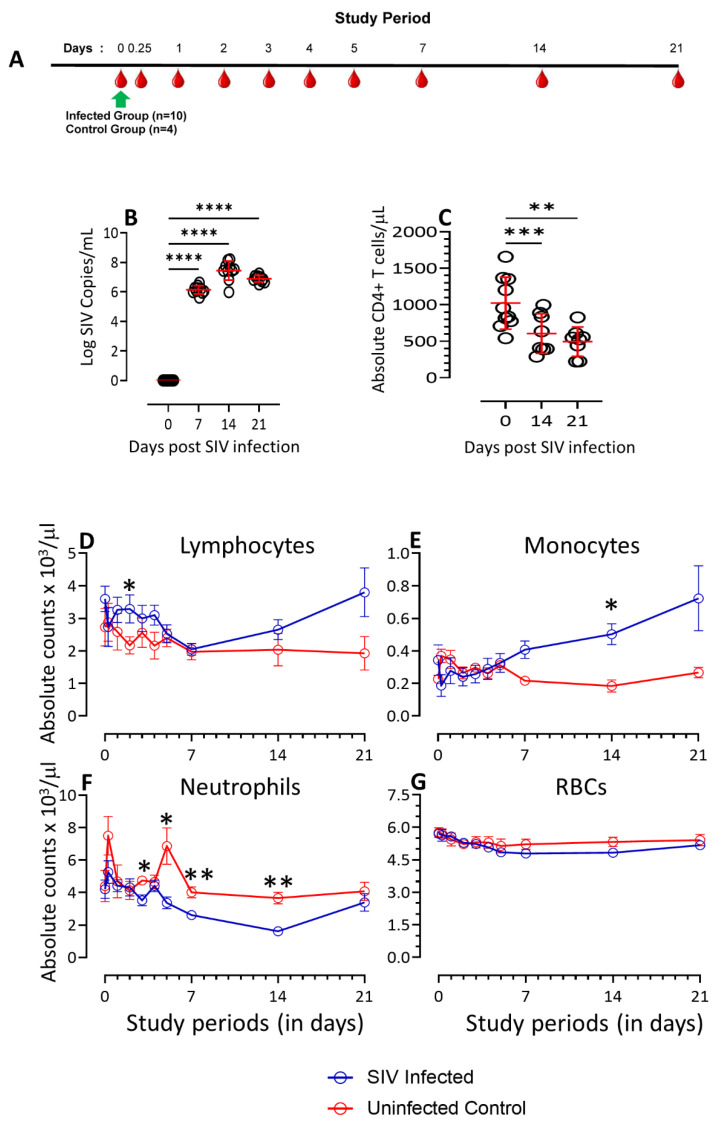
Animal schedule, plasma viral load (PVL), peripheral absolute CD4, lymphocytes, monocytes, neutrophils, and red blood cell (RBC) count are shown. (**A**) Schematic representation of the animal experiment using 10 SIV-infected and 4 control macaques. Note that the SIV-infected macaques were challenged at day 0 time point (green arrow). The red blood drop symbols indicate sample collection time points in days. (**B**) PVL with mean ± SE in macaques during the acute phase of SIV infection, as determined using RT-PCR (*n* = 10). (**C**) Absolute counts of peripheral CD4+ T cells at pre- and post-SIV infection. Each point of the scattered plot represents data from individual macaques. Asterisks indicate statistical differences between time points as calculated using Dunnett’s multiple comparison analysis (**, *p* ≤ 0.01; ***, *p* ≤ 0.001; ****, *p* ≤ 0.0001). Absolute counts of lymphocytes (**D**), monocytes (**E**), neutrophils (**F**), and RBCs (**G**) in peripheral blood of SIV infected (*n* = 10) and uninfected control (*n* = 4) macaques are shown. Data presented as mean ± SE of different cell populations for the entire 21 days study period. Mann–Whitney *t*-test analysis was used to determine statistically significant differences between SIV-infected and uninfected-control groups in all those cell populations for that specific time (*, *p* ≤ 0.05; **, *p* ≤ 0.01).

This study used 14 RhMs of both sexes (six females and eight males) between 6.3 and 8.3 years of age. The macaques were seronegative for SIV, simian T-cell leukemia virus type 1, and type-D retroviruses antibodies at the initiation of the study. The subjects were randomly assigned into two groups (infected and uninfected control), with ten and four RhMs per group. The blood collected from all the SIV-infected RhMs was studied longitudinally for 21 days post-SIV infection for hematology, virological, and immunological assays (Figure 1A). The macaques from the infected group were inoculated with 100 TCID_50_ pathogenic SIV_MAC_251 using an intravenous route to mimic one of the primary routes of HIV transmission in humans. The blood was also collected from all the uninfected controls for all those longitudinal time points to perform hematology and immunological assays (Figure 1A). For analysis, plasma and serum samples were collected at various time points, including 0, 0.25, 1, 2, 3, 4, 5, 7, 14, and 21 days post-infection (dpi) or study periods (Figure 1A).

### 2.3. Hematology

Hematology was performed on EDTA-anticoagulated blood using a Sysmex XT2000i analyzer (Sysmex Corporation, Kobe, Japan). Fresh blood was used for all the hematology analyses.

### 2.4. Plasma Isolation

The plasma was isolated from EDTA-anticoagulated blood after centrifugation. The frozen plasma samples were used for plasma viral load and cytokine/chemokine analysis.

### 2.5. Quantitative SIV Plasma Virus Load (PVL)

Quantitative reverse transcription-PCR (qRT-PCR) was used to measure plasma viral RNA copies at the Wisconsin National Primate Research Center with a lower detection limit of 60 SIV RNA copies/mL of plasma [9].

### 2.6. Quantification of Cytokines and Chemokines in Plasma

Sixty-one cytokines/chemokines in plasma were quantified using the U-plex biomarker NHP 61 plex (Meso Scale Diagnostics; MSD, MD, USA) to determine changes in CTACK (C-c motif chemokine ligand 27; CCL27), eotaxin-1 (CCL11), eotaxin-2 (CCL24), eotaxin-3 (CCL26), ENA-78, Fractalkine, FLT3L (FMS-like tyrosine kinase 3 ligand), G-CSF (granulocyte colony-stimulating factor), GM-CSF (granulocyte-macrophage colony-stimulating factor), I-309 (CCL1), GRO-α (CXCL1), IFN-α2a, IFN-γ, IL-1α (interleukin-1α), IL-1β, IL-1RA (interleukin-1 receptor antagonist), IL-2, IL-2Rα, IL-4, IL-5, IL-6, IL-7, IL-8 (CXCL8), IL-9, IL-10, IL-12, IL-12p70, IL-13, IL-15, IL-16, IL-17A, IL-17A/F, IL-17B, IL-17C, IL-17D, IL-17F, IL-18, IL-22, IL-23, IP-10 (IFNγ inducible protein 10; CXCL10), I-TAC (Interferon-inducible T-cell alpha chemoattractant; CXCL11), MCP-1 (monocyte chemotactic protein-1; CCL2), MCP-2 (CCL8), MCP-3 (CCL7), MCP-4 (CCL13), M-CSF (macrophage colony-stimulating factor), MDC (macrophage-derived chemokine; CCL22), MIF (macrophage migration inhibition factor), MIP-1α (macrophage inflammatory protein 1α; CCL3), MIP-1β (CCL4), MIP-3α (CCL20), MIP-3β (CCL19), MIP-5 (CCL15), SDF-1α (stromal cell-derived factor-1 alpha; CXCL12), TARC (thymus and activation regulated chemokine), TNF-α (tumor necrosis factor-α), TNF-β, TPO (thrombopoietin), TRAIL (TNF-related apoptosis-inducing ligand), VEGF-α (vascular endothelial growth factor-α), and YKL-40 (chitinase-3-like protein 1) following the manufacturer instruction with minor modification [8]. U-plex plates were coated with respective biotinylated capture antibodies and incubated overnight on a shaker at 4 °C. Calibrator standards and diluted plasma samples were added to the individual wells after washing with wash buffer. The plate was incubated overnight on a shaker at 4 °C. The next day, the plate was washed with wash buffer and the detection antibody was added to the well and incubated on a shaker at room temperature for 1 h. The plate was finally washed and a read buffer was added. The plate was read immediately on an MSD microplate reader (MSD). The concentration of each cytokine and chemokine was determined based on the calibration standard curve and its respective signals.

### 2.7. Flow Cytometry of Peripheral Blood Mononuclear Cells (PBMCs)

The PBMCs were isolated from whole heparinized blood using density gradient centrifugation [10,11]. The CD4 and CD8 T cell frequencies were quantified in the PBMCs using directly conjugated monoclonal antibodies. Anti-CD45 (clone D058-1283), anti-CD3 (clone SP34.2), anti-CD4 (clone L200), and anti-CD8 (clone SK1) monoclonal antibodies obtained from BD Biosciences were used for staining. Live/Dead stain (Thermo Fisher Scientific, Waltham, MA, USA) was used to exclude dead cells from the flow analysis. After surface staining and fixation, the cells were acquired on a Becton Dickinson LSRII instrument. At least 50,000 events were acquired from each sample using lymphocyte gating and analyzed using FlowJo software (v10.8, BD Biosciences, Franklin Lakes, NJ, USA). The absolute CD4 count was calculated using complete blood count (CBC) data from hematology and flow cytometry CD4+ T cells percentage analysis.

### 2.8. Statistical Analyses

GraphPad Prism was used for all the statistical analysis and generating graphs (v9.4.1., GraphPad Software, San Diego, CA, USA). One-way repeated ANOVA measured significant differences between post-SIV time points and baseline data. Dunnett’s multiple comparison tests were applied to examine any statistically significant changes in the cytokine/chemokine level at different post-infection time points compared to the baseline 0 day or pre-infection. A Mann–Whitney *t*-test as a nonparametric method was performed to determine the statistical differences between the various blood cell counts of the SIV-infected and the uninfected control group. A correlation analysis between PVL and the significantly upregulated or downregulated cytokine/chemokine expression during acute SIV infection and correlation analysis between the CD4+ T cell count and cytokine/chemokine level expressions were performed using the two-tailed Pearson correlation method. A *p*-value of < 0.05 was considered statistically significant throughout the analysis.

## 3. Results

### 3.1. Dynamics of PVL, CD4, Lymphocytes, Monocytes, Neutrophils, and Red Blood Cells Count in SIV-Infected Macaques

Ten SIV-infected RhMs were used to evaluate the cytokine and chemokine profile during the early 21 days of the infection. Since the blood samples were drawn very often during the initial stage of this study, we have also studied four healthy, SIV-uninfected RhMs as a control to determine whether the changes detected in the SIV-infected RhMs are not due to the results of repetitive blood collection. All 10 SIV-infected RhMs remained SIV-infected throughout this study. The mean plasma viral load significantly increased at day 7 (log 10^6.1^ copies/mL of plasma, *p* ≤ 0.0001) and peaked at day 14 post-SIV infection (10^7.4^, *p* ≤ 0.0001) (Figure 1B). Similar to PVL, the peripheral CD4 count also significantly decreased at day 14 (mean ± SE, 605 ± 96 cells/µL of blood, *p* ≤ 0.001) compared to the baseline (1022 ± 112 cells/µL of blood) and the count remained lower at 21 days post-SIV infection (Figure 1C). We were unable to detect any significant difference in the PVL and cell counts between the male and female SIV-infected RhMs. The CD4+ T cell count in uninfected control RhMs remained normal throughout the study.

The lymphocyte population in both the SIV-infected and control RhMs remained within the range of 1.97−3.80 × 10^3^/µL of blood. The lymphocyte counts remained stable primarily between groups except on day 2 (*p* = 0.048, Figure 1D), when the lymphocyte count was significantly elevated in the SIV-infected RhMs compared to the controls. Similarly, the monocyte count ranged between 0.09−0.82 × 10^3^/µL of blood. The monocyte count increased dramatically at the day 14 time point (*p* = 0.019, Figure 1E) in the SIV-infected RhMs compared to the controls. The neutrophil absolute counts decreased in the SIV-infected RhMs at 3- (*p* = 0.002), 5- (*p* = 0.011), 7- (*p* = 0.008), and 14- (*p* = 0.002, Figure 1F) day study periods when compared to the control group. No significant changes were detected in the red blood cell (RBC) counts between those two groups (Figure 1G).

### 3.2. Cytokine Profiling during Acute SIV Infection

**Figure 2:**
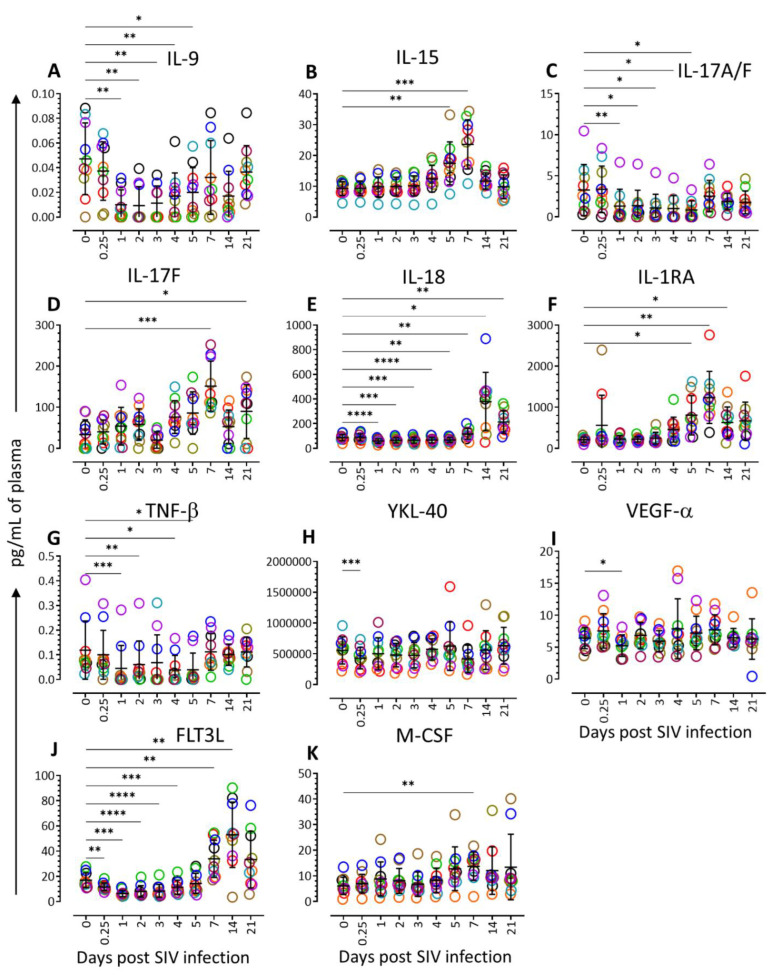
Plasma cytokine profile during early acute SIV infection. Scattered plot with mean ± SE for 11 different cytokine ((**A**), IL-9; (**B**), IL-15; (**C**), IL-17A/F; (**D**), IL-17F; (**E**), IL-18; (**F**), IL-1RA; (**G**), TNF-β; (**H**), YKL-40; (**I**), VEGF-α; (**J**), FLT3L; and (**K**), M-CSF) responses observed throughout the SIV infection time points are shown. Each point on the scattered plot denoted by different color represents the cytokine concentration for an individual macaque. Asterisks indicate statistical differences between day 0 and post-SIV infection time points as calculated using Dunnett’s multiple comparison analysis (*, *p* ≤ 0.05; **, *p* ≤ 0.01; ***, *p* ≤ 0.001; ****, *p* ≤ 0.0001).

**Figure 3:**
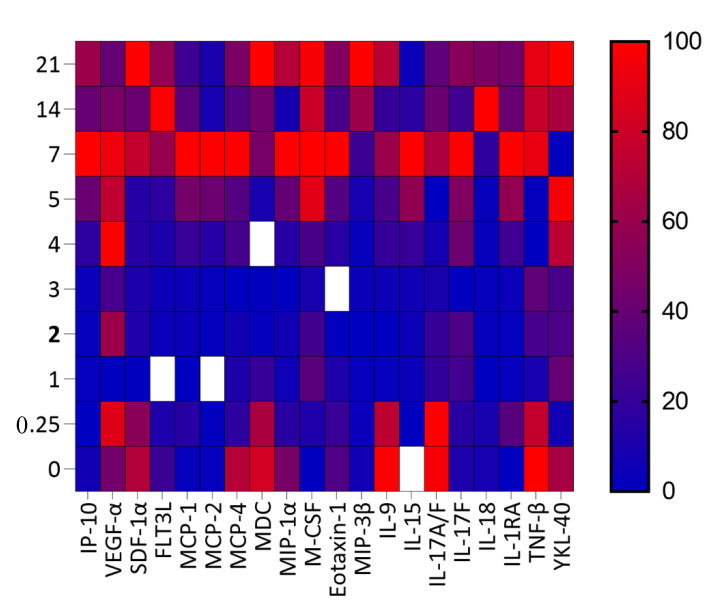
Heat mapping of 20 essential cytokines/chemokines with continuous color shading over 21 days post-SIV infection (*n* = 10). Cytokines/chemokines are ordered from left to right on the X-axis and post-SIV infection time points are shown in days on the Y-axis. Most cytokine/chemokine levels remained low on days 2–3 post-SIV infection. The cytokine/chemokine level increased at day 5 and the majority remained high up to day 21 post-SIV infection.

The plasma cytokine and chemokine levels were quantified from all 14 RhMs for all the time points. Out of a total of 61 different cytokines and chemokines, we noted that the level of 11 cytokines (IL-9, IL-15, IL-17A/F, IL-17F, IL-18, IL-1RA, TNF-β, YKL-40, VEGF-α, FLT3L, and M-CSF) was significantly altered at one or more time points when compared to the baseline (Figure 2). The Plasma IL-9, a growth factor and antiapoptotic cytokine that is primarily expressed by activated T cells, were significantly decreased at 1 dpi (mean ± SE; 0.010 ± 0.004, *p* = 0.004), 2 dpi (0.009 ± 0.005, *p* = 0.003), 3 dpi (0.011 ± 0.005 pg/mL, *p* = 0.003), 4 dpi (0.017 ± 0.006, *p* = 0.008), and 5 dpi (0.020 ± 0.006, *p* = 0.017) compared to the pre-infection time point (0.047 ± 0.009). However, after day 5, no significant changes in IL-9 levels were detected for the later time points when compared to the baseline values (7 dpi: 0.032 ± 0.009, *p* = 0.232; 14 dpi: 0.017 ± 0.006, *p* = 0.059; 21 dpi: 0.036 ± 0.007, *p* = 0.736) (Figure 2A and Figure 3). Interestingly, we have detected a significant increase in plasma IL-15 level as early as 5 dpi (17.450 ± 2.205 pg/mL, *p* = 0.008) and 7 dpi (23.610 ± 2.517 pg/mL, *p* = 0.0005) when compared to the baseline (9.314 ± 0.702 pg/mL). The IL-15 plasma concentration returned to normal at days 14 and 21 dpi (9.838 ± 1.216 pg/mL) (Figure 2B and Figure 3). IL-15 is a positive regulator of cellular processes such as cell maturation and the activation of T and NK cells.

The Plasma IL-17A/F complex was found to be decreased significantly from 1 to 5 dpi (1 dpi: 1.3 ± 0.6 pg/mL, *p* = 0.006; 2 dpi: 1.4 ± 0.6, *p* = 0.015; 3 dpi: 1.1 ± 0.5, *p* = 0.014; 4 dpi: 1.0 ± 0.5, *p* = 0.014; and 5 dpi: 0.8 ± 0.3, *p* = 0.035) compared to baseline (3.3 ± 1.0 pg/mL). After 5 dpi, the level returned to normal for the rest of the study time points (Figure 2C and Figure 3). In contrast, the level of IL-17F, an inflammatory cytokine, was significantly increased at 7 dpi (151.1 ± 19.3 pg/mL, *p* = 0.0003) and 21 dpi (89.7 ± 20.6, *p* = 0.0187) compared to the pre-infection (33.7 ± 11.2 pg/mL) time point (Figure 2D and Figure 3).

IL-18, a proinflammatory cytokine that can modulate both innate and adaptive immunity [12], decreased significantly from 1 to 5 dpi (1 dpi: 60.5 ± 5.7 pg/mL, *p* < 0.0001; 2 dpi: 66.0 ± 6.5, *p* = 0.0002; 3 dpi: 66.3 ± 7.6, *p* = 0.001; 4 dpi: 68.3 ± 7.1, *p* < 0.0001; and 5 dpi: 68.8 ± 6.5, *p* = 0.001) compared to baseline (87.9 ± 8.2 pg/mL) (Figure 2E and Figure 3). However, after 5 dpi, the IL-18 level increased significantly at 7 dpi (117.4 ± 13.6 pg/mL, *p* = 0.008), 14 dpi (378.4 ±74.9 pg/mL, *p* = 0.015), and 21 dpi (212.9 ± 28.6, *p* = 0.007) suggesting that IL-18 has a significant role in viral replication and disease progression from day 7 onwards.

IL-1RA, the receptor protein for IL-1A, IL-1B, and IL-1R1, was significantly upregulated in plasma during 5 dpi (804.2 ± 151.1 pg/mL, *p* = 0.013), 7 dpi (1235.6 ± 200.7, *p* = 0.004), and 14 dpi (625.9 ± 119.6, *p* = 0.032) compared to the baseline (211.4 ± 19.3 pg/mL) (Figure 2F and Figure 3). The IL-1RA level was also high at 21 dpi (664.9 ± 144.2 pg/mL, *p* = 0.067) compared to the baseline, but the value was not statistically significant.

TNF-β, a cytokine produced by lymphocytes responsible for immune stimulation and antiviral responses, was downregulated at the early stage of SIV infection. The TNF-β level was significantly low at 1 dpi (0.04 ± 0.03 pg/mL, *p* = 0.0004), 2 dpi (0.06 ± 0.03 pg/mL, *p* = 0.002), 4 dpi (0.04 ± 0.02 pg/mL, *p* = 0.017), and 5 dpi (0.04 ± 0.02 pg/mL, *p* = 0.021) compared to the pre-infection (0.12 ± 0.04 g/mL) (Figure 2G and Figure 3). The cytokine levels reverted to near normal at 7 dpi onwards (Table S1). YKL-40, a proinflammatory cytokine produced by activated macrophages, neutrophils, synovial cells, and chondrocytes, contributes to inflammatory disease progression, fibrosis, and poor prognosis for different cancers [13,14]. There was no significant change in the YKL-40 levels at any time, except at 6 h post-infection (431,334 ± 55,505 pg/mL, *p* = 0.0004) when the concentrations were significantly lower compared to the baseline (556,552 ± 69,555 pg/mL; Figure 2H and Figure 3).

VEGF-α, an essential inducer for angiogenesis [15], remained normal throughout the time point of this study except at 1 dpi (5.2 ± 0.5 pg/mL, *p* = 0.011), where the VEGF-α concentration significantly reduced compared to the baseline (6.4 ± 0.5 pg/mL; Figure 2I and Figure 3). FLT3L plays a significant role in the proliferation and development of natural killer cells and dendritic cells [16]. Initially, the plasma FLT3L level was significantly downregulated at 6 h (11.7 ± 1.0 pg/mL, *p* = 0.006), 1 dpi (6.4 ± 0.8 pg/mL, *p* = 0.0002), 2 dpi (8.1 ± 1.5 pg/mL, *p* < 0.0001), 3 dpi (8.5 ± 1.6 pg/mL, *p* < 0.0001), and 4 dpi (11.4 ± 1.8 pg/mL, *p* = 0.0005), compared to the pre-infection (17.0 ± 1.8 pg/mL) time point. However, the FLT3L level increased significantly at 7 dpi (33.9 ± 4.6 pg/mL, *p* = 0.006) and 14 dpi (53.0 ± 8.2 pg/mL, *p* = 0.003) compared to baseline values (Figure 2J and Figure 3). The FLT3L level remained high at 21 dpi (33.29 ± 7.08, *p* = 0.09) but remained nonsignificant compared to the baseline. A significant upregulation of M-CSF expression was also detected at 7 dpi (13.7 ± 1.7 pg/mL, *p* = 0.002) compared to the baseline data (6.1 ± 1.1 pg/mL). No difference in the M-CSF level was detected for any other time points compared to baseline (Figure 2K and Figure 3).

Several other cytokines, including IFN-γ, IL-1α, IL-1β, IL-2, IL-4, IL-5, IL-6, IL-7, IL-8, IL-10, IL-12, IL-12p70, IL-13, IL-15, IL-16, IL-17A, IL-17B, IL-17C, IL-17D, IL-22, IL-23, and TNF-α were detectable during the early acute time points. However, no significant changes were observed at any time compared to the baseline (Table S1). We were also unable to see any significant differences in any of the cytokine levels from SIV-uninfected controls for any of the time points compared to the day 0 time point (Figure S1 and Table S1).

### 3.3. Chemokine Profiling during Acute SIV Infection

**Figure 4:**
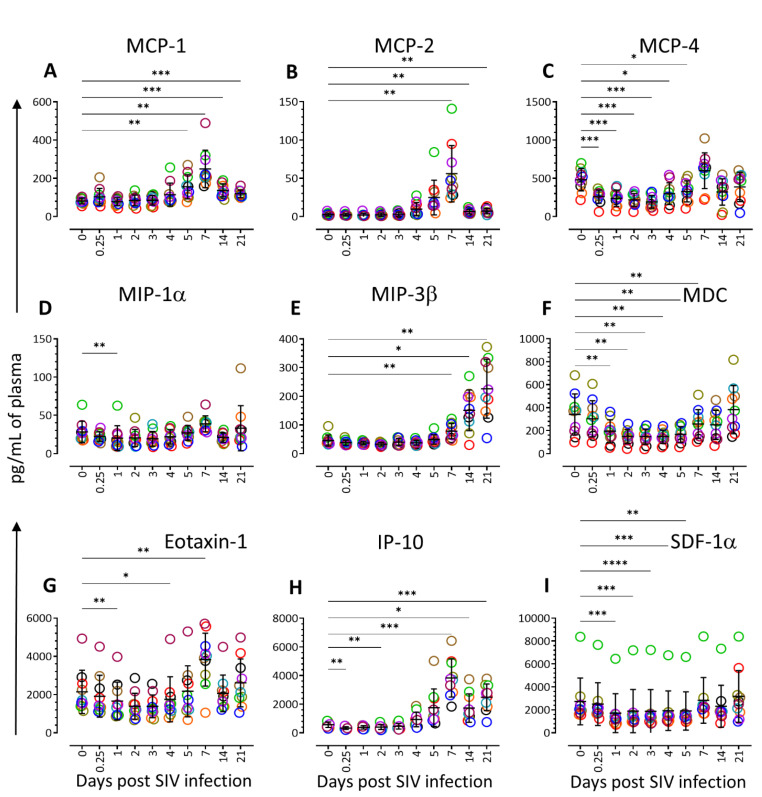
Plasma chemokine profile during early acute SIV infection. Scattered plot with mean ± SE for nine different chemokine ((**A**), MCP-1; (**B**), MCP-2; (**C**), MCP-4; (**D**), MIP-1α; (**E**), MIP-3β; (**F**), MDC; (**G**), Eotaxin-1; (**H**), IP-10; and (**I**), SDF-1α) responses observed throughout the SIV infection time points are shown. Each point of the scattered plot denoted by different color represents the chemokine concentration for an individual macaque. Asterisks indicate statistical differences between day 0 and post-SIV infection time points as calculated using Dunnett’s multiple comparison analysis (*, *p* ≤ 0.05; **, *p* ≤ 0.01; ***, *p* ≤ 0.001; ****, *p* ≤ 0.0001).

Similar to the cytokines, we have also observed significant changes in nine chemokines (MCP-1, -2, -4, MIP-1α, -3β, MDC, Eotaxin-1, IP-10, and SDF-1α) when compared to the pre-infection time point (Figure 3 and Figure 4). The MCP-1 and MCP-2 levels significantly increased after SIV infection compared to the baseline (Figure 4A,B). A significant increase in MCP-1 expression was detected at 5 dpi (156.1 ± 20.0 pg/mL, *p* = 0.008), 7 dpi (249.4 ± 30.9, *p* = 0.002), 14 dpi (137.1 ± 10.8 pg/mL, *p* = 0.0008), and 21 dpi (119.2 ± 6.2 pg/mL, *p* = 0.0002) compared to day 0 (81.4 ± 4.9). MCP-2 was also significantly upregulated at 7 dpi (56.1 ± 11.6 pg/mL, *p* = 0.007), 14 dpi (6.5 ± 1.0, *p* = 0.005), and 21 dpi (7.2 ± 1.1, *p* = 0.010) compared to day 0 (2.7 ± 0.6 pg/mL) (Figure 3 and Figure 4B). Similar to FLT3L, the expression of MCP-4 was also significantly downregulated at 1 dpi (239.7 ± 33.9, *p* = 0.0001), 2 dpi (218.3 ± 26.3, *p* = 0.0007), 3 dpi (192.9 ± 26.6, *p* = 0.0001), 4 dpi (300.3 ± 44.9, *p* = 0.035), and 5 dpi (328.8 ± 43.2, *p* = 0.013) compared to day 0 (481.8 ± 46.4). The MCP-4 level increased significantly at 7 dpi and remained normal for the rest of the period (Figure 3 and Figure 4C). A significant reduction in the MIP-1α level was detected only at 1 dpi compared to the baseline (20.2 ± 5.1 and 28.3 ± 4.4 for 1 dpi and day 0, respectively, *p* = 0.007) (Figure 3 and Figure 4D). In contrast, the plasma MIP-3β level increased significantly at 7 dpi (78.4 ± 8.4 pg/mL, *p* = 0.005), 14 dpi (150.9 ± 22.4, *p* = 0.011), and 21 dpi (226.2 ± 32.5, *p* = 0.001) compared to the day 0 (46.3 ± 6.0, Figure 3 and Figure 4E).

The plasma MDC level remained significantly lower at 1 dpi (194.6 ± 31.4 pg/mL, *p* = 0.009), 2 dpi (149.7 ± 18.9, *p* = 0.008), 3 dpi (148.1 ± 20.7, *p* = 0.005), 4 dpi (147.2 ± 17.9, *p* = 0.01), 5 dpi (166.8 ± 21.9, *p* = 0.008), and 7 dpi (256.6 ± 39.8, *p* = 0.009). The expression returned to normal for the rest of the time points (Figure 3 and Figure 4F, and Table S1). Similar to MDC, the Eotaxin-1 level was also significantly decreased at 1 dpi (1659.0 ± 313.5 pg/mL, *p* = 0.008) and 4 dpi (1750.6 ± 372.6, *p* = 0.036) when compared to baseline (2144.8 ± 357.9, Figure 4G). The Eotaxin-1 level again significantly increased at 7 dpi (3829.8 ± 436.5, *p* = 0.005) compared to day 0 time point.

The plasma IP-10 concentration was significantly downregulated at 6 h (340.5 ± 30.3 pg/mL, *p* = 0.006), 2 dpi (405.7 ± 52.5, *p* = 0.007) compared to the pre-infection time point (570.6 ± 61.4) (Figure 4H and Table S1). The IP-10 level increased after that and was found significantly upregulated at 7 dpi (3831.7 ± 420.3, *p* = 0.0001), 14 dpi (1734.2 ± 312.5, *p* = 0.016), and 21 dpi (2485.2 ± 291.7, *p* = 0.0002).

In contrast to IP-10, a significant downregulation of SDF-1α expression was detected during the early SIV infection. The SDF-1α level was significantly downregulated at 1 dpi (1719.7 ± 535.8 pg/mL, *p* = 0.0002), 2 dpi (1895.1 ± 596.3, *p* = 0.0002), 3 dpi (1870.8 ± 602.6, *p* < 0.0001), 4 dpi (1930.7 ± 543.2, *p* = 0.0009), and 5 dpi (1910.9 ± 528.3, *p* = 0.0020) compared to day 0 (2734.1 ± 44.9). The SDF-1α level returned to normal from day 7 post-SIV infection onwards (Figure 3 and Figure 4I).

Several other chemokines, including CTACK, eotaxin-2, eotaxin-3, I-TAC, MIP-1β, GRO-α, MIP-3α, and MIP-5, were also evaluated during the early acute time points. However, no significant changes were detected at any time compared to the baseline (Table S1). We also did not see any substantial differences in chemokine levels from SIV-uninfected control macaques for any time points compared to day 0 (Figure S1 and Table S1).

### 3.4. Correlation of Cytokines/Chemokines Concentration with PVL and Absolute CD4+ T Cell Count

**Figure 5:**
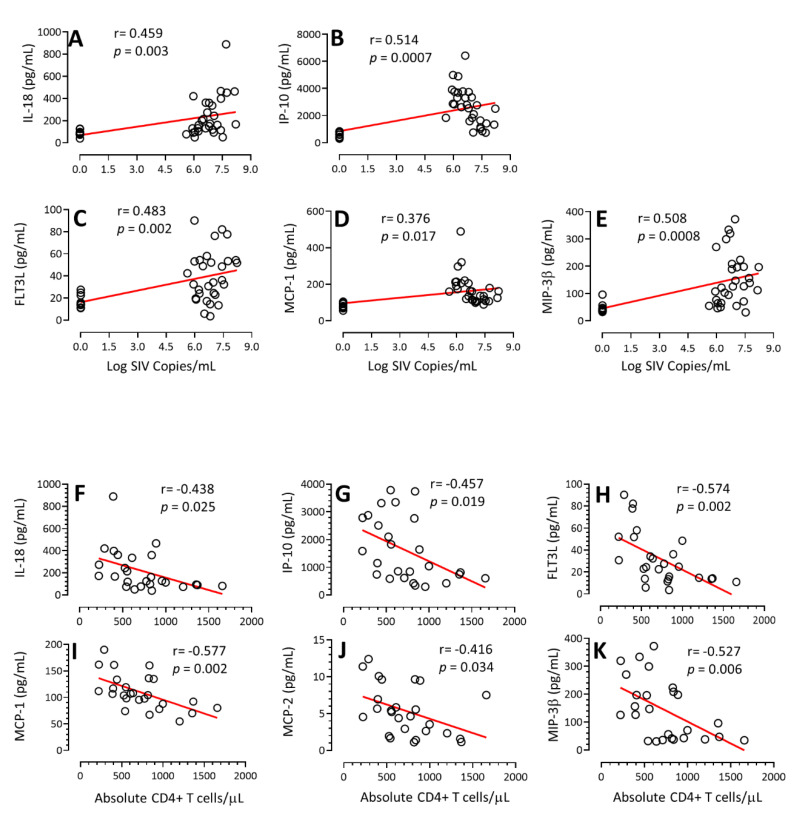
Correlation of cytokine/chemokine with plasma viral load (PVL) and absolute peripheral CD4+ T cell count. Spearman’s rank correlation coefficient of determination between IL-18 and PVL (**A**), IP-10 and PVL (**B**), FLT3L and PVL (**C**), MCP-1 and PVL (**D**), and MIP-3β and PVL (**E**) is shown for all SIV-infected macaques. A significant positive correlation was detected with the expression of IL-18, IP-10, FLT3L, MCP-1, and MIP-3β with PVL during SIV infection. Spearman’s rank correlation analysis between IL-18 and CD4 count (**F**), IP-10 and CD4 count (**G**), FLT3L and CD4 (**H**), MCP-1 and CD4 (**I**), MCP-2 and CD4 count (**J**), and MIP-3β and CD4 count (**K**) showed a significant negative correlation with the increase in IL-18, IP-10, FLT3L, MCP-1, MCP-2, and MIP-3β and loss of peripheral CD4+ T cells (*n* = 10). The correlation and significant values are shown for each plot. Black open circles represent pre and post SIV infection time point from each SIV infected macaque. Red lines represent simple linear regression for each plot.

To determine if the cytokine/chemokine responses detected early during SIV infection correlated with increased PVL and loss of peripheral CD4+ T cell count, we have performed correlation coefficient analysis between PVL and each of the significantly upregulated or downregulated cytokines/chemokines during acute SIV infection. A significant positive correlation was observed between IL-18 and PVL (r = 0.46, *p* = 0.003, Figure 5A), IP-10 and PVL (r = 0.51, *p* = 0.0007, Figure 5B), FLT3L and PVL (r = 0.48, *p* = 0.002, Figure 5C), MCP-1 and PVL (r = 0.38, *p* = 0.017, Figure 5D), and MIP-3β and PVL (r = 0.51, *p* = 0.0008, Figure 5E) suggesting that either increased PVL has an impact on upregulating the expression of those cytokines/chemokines or the upregulation of cytokines/chemokines had a significant role in inducing more viral replication. Similarly, a Pearson’s correlation analysis between the CD4+ T cell count and cytokine/chemokine levels demonstrated a negative correlation between IL-18 and CD4 (r = −0.44, *p* = 0.025, Figure 5F), IP-10 and CD4 (r = −0.46, *p* = 0.019, Figure 5G), FLT3L and CD4 (r = −0.57, *p* = 0.002, Figure 5H), MCP-1 and CD4 (r = −0.58, *p* = 0.002, Figure 5I), MCP-2 and CD4 (r = −0.42, *p* = 0.034, Figure 5J), and MIP-3β and CD4 (r = −0.53, *p* = 0.006, Figure 5K) suggesting that increased cytokine/chemokine responses have a significant impact on CD4 T cell loss as well as increased viral replication.

## 4. Discussion

Tightly regulated cytokine and chemokine production are ideal for an effective antiviral immune response. The virus-induced increased cytokine/chemokine responses are associated with diseases such as influenza, severe acute respiratory syndrome coronavirus 2 (SARS-CoV-2), SARS-CoV, and Middle East respiratory syndrome coronavirus [17]. The association of CD4+ T cell loss, increased viral load, and viral set-point with cytokine storm during HIV/SIV infection was well documented earlier [3,4,5,6,18]. The dysregulation of cytokines after HIV/SIV infection was also reported to be associated with various clinical events such as comorbidities, disease progression, and mortality [19,20].

Multiple HIV infection studies have shown changes in systemic plasma cytokine/chemokine levels, including IFN-α, TNF-α, IFN-γ, IL-2, IL-4, IL-10, IP-10, and IL-1RA during the acute phase [2,21,22,23,24,25]. However, a significant limitation of all these human studies was the shortage of information about the exact time of initial HIV infection. Similarly, studies with acute SIV infection have been performed in non-human primates by measuring cytokine mRNA expressions in tissues [26,27,28,29,30,31,32,33,34]. The changes in MIP-1α, TNF-α, IL-6, and IFN-α were detected in different mucosal and LN tissues at 3–10 days post-mucosally infected macaques [32]. Increased MIP-3α mRNA expression has also been reported in endocervical epithelium 24 h after mucosal SIV infection [33]. Conversely, the relationship between the abundance of mRNA expression and the manifestation of cellular protein levels is complex. The mRNA transcript levels are insufficient to determine the predicted protein levels as it depends on the cells’ steady or highly dynamic state and the post-transcriptional and post-translational regulation [35,36,37]. Immunohistochemistry analysis showed increased TGF-β in LN tissue expression at day 7 from a cohort of female RhMs infected intravenously with 1 MID_50_ SIV_MAC_239 [38]. Our earlier study using flow cytometry and immunohistochemistry assays showed a significant increase in TGF-β expression in intestinal mucosal tissue at 21 days post-SIV infection [39]. Nevertheless, a clear understanding of the regulation of different cytokines/chemokines during acute SIV infection is needed when the most sensitive and robust MSD technology [40] allows us to measure multiple cytokines/chemokines in circulation. The SIV-RhM model strongly recapitulated the HIV infection in humans and was used here to understand the dynamics of 61 different cytokines and chemokines involved in controlling immune cell trafficking and regulating the nature of immune responses during acute infection. We observed a significant change in 11 cytokines/growth factors and 9 chemokines during acute SIV infection. The deviations in the amounts were detected as early as 6 h post-infection and were below the baseline values in all the instances for at least four days post-infection.

IL-15 upregulates early during SIV/HIV infection, which concurs with our findings and impacts increasing CD4 expression on memory CD4+ T cells [21,41]. However, we did not find any correlation between IL-15 and PVL or CD4 counts. IL-1RA plays an essential role in immune regulation by inhibiting proinflammatory cytokines such as IL-1α and IL-1β [42]. Even though nearly the same stimuli induce IL-1RA and IL-1, increased expression of IL-1RA and no change in the IL-1β production was observed. IL-1RA production was also described to be correlated with the markers of HIV progression [43]. Increased circulatory IL-18 is thought to contribute to HIV pathogenesis by enhancing the death of NK cells, as observed in *in vitro* studies [44]. A significant correlation between increased circulatory IL-18 and decreased CD4 count suggests that IL-18 can be considered a potential biomarker of disease progression at the acute stage of infection. IL-18 was also reported to promote HIV replication by upregulating the CXCR4 expression [12]. Our data confirm that IP-10 can also be a potential biomarker to predict PVL and CD4 levels during acute infection. An earlier *in vitro* study with PBMCs and monocyte-derived macrophages demonstrated that IP-10 induces increased HIV-1 replication [45] by dysregulating T- and NK-cell functions [46,47]. Increased IP-10 after HIV infection and its contribution to disease progression have been reported elsewhere [21,48,49,50]. FLT3L has been reported as a protective cytokine in reducing viremia by expanding and mobilizing the plasmacytoid dendritic cells (pDCs) during acute HIV infection in a humanized mice model [51]. However, the significant correlation between FLT3L and PVL or CD4 levels suggests that increased FLT3L is fueling more viral replication by enhancing lymphopoiesis. It is also possible that protective FLT3L is trying to improve the pDC population, but those cells are not functional enough to control the CD4+ T cell loss and viral replication.

MCP-1 upregulates CXCR4 coreceptor expression in resting CD4+ T cells, facilitated CXCR4-tropic HIV to infect the CD4+ T cells [52], and was associated with CNS disorder [53]. Here, we have also shown a significant correlation between MCP-1 and PVL or CD4 levels suggesting that MCP-1 can be used as a biomarker to demonstrate acute SIV pathogenesis and disease progression, which agrees with previous studies [54,55]. MCP-2/MCP-4/MIP-1α and Eotaxin-1 bind to CCR5 and CCR3, respectively, and regulate viral entry to the target cells [56,57]. MCP-2 is also involved in monocyte migration and inflammatory responses and has been recently shown to be involved in acute respiratory distress syndrome [58,59,60]. Increased MCP-2 expression during acute infection might have a significant role in inflammation and the inhibition of MCP-2 expression might be beneficial in controlling early inflammation, which needs future study.

MIP-3β facilitates HIV entry and the establishment of latency by allowing the integration of viral genomes in resting CD4+ T cells from *in vitro* studies [61,62]. Our study also suggests that MIP-3β can be used as an additional biomarker to determine the viral replication and the loss of CD4+ T cells during acute infection. Some dysregulated chemokines regulate the HIV infection or replication by targeting virus co-receptors directly. These chemokines include SDF-1α, MCP-2, MCP-4, MIP-1α, and Eotaxin-1, which bind either to CXCR4, CXCR5, CCR5, or CCR3 and regulate viral infection [63]. The downregulation of SDF-1α in our study might indicate that SIV tends to infect CD4+ T cells more efficiently due to the availability of CXCR4 receptors at the early stage of infection.

Neutrophils are the first cells responsible for primary defense against pathogens and regulating inflammation and immune functions [64]. Neutropenia develops in HIV-infected individuals at the chronic stage of the disease. However, neutropenia has also been reported during acute infection [65,66], which is in agreement with our data suggesting that neutropenia might have a significant effect in inducing primary defense mechanisms as well as downregulations of several major cytokines/chemokines during the early few days after infection, which leads to increased viral replication and CD4 T cell loss.

## 5. Conclusions

The loss of peripheral CD4 T cells and increased PVL detected at day 14 and day 7 post-SIV infection, respectively, as well as neutropenia seen from day 3 to day 14 in SIV-infected RhMs, suggest that the early loss of neutrophil-mediated primary defense has a significant impact on initial virus replication and the loss of peripheral CD4+ T cells. A substantial change in 11 cytokines/growth factors and 9 chemokines out of 61 cytokines/chemokines were observed during acute SIV infection. Almost all the cytokines/chemokines were below the baseline values for an initial couple of days of infection. Our experimental design does not address any responses detected beyond the 21 days of acute infection. Since we have only quantified cytokines and chemokines and performed the correlation analysis, the role of each cytokine/chemokine in modulating disease progression requires future study. We detected six important cytokines/chemokines (IL-18, IP-10, FLT3L, MCP-1, MCP-2, and MIP-3β) that can be used as biomarkers to predict the peripheral CD4+ T cell loss and increased viral replication during the acute phase of SIV/HIV infection. Hence, vaccines or drugs modulating IL-18, IP-10, FLT3L, MCP-1, MCP-2, and MIP-3β expression might be promising novel tools to induce early antiviral responses to combat acute SIV/HIV infection.

## Data Availability

All relevant data are included within the manuscript. The raw data are available on request from the corresponding author.

## Supplementary Materials

The following supporting information can be downloaded at: https://www.mdpi.com/article/10.3390/vaccines11020264/s1, Figure S1: Twenty cytokine/chemokine profiles from plasma for a 21-day study period in uninfected control macaques are shown (*n* = 4). Each red circle of the scattered plot (including mean ± SE) represents cytokine/chemokine concentration for an individual macaque. There was no significant difference in the cytokine/chemokine values at any time point compared to day 0.
