# Supplementary material for: Divergent Cytokine and Chemokine Responses at Early Acute Simian Immunodeficiency Virus Infection Correlated with Virus Replication and CD4 T Cell Loss in a Rhesus Macaque Model
Source: Vaccines (Basel). 2023 Jan 25;11(2):264. doi: 10.3390/vaccines11020264 (PMC9963901; doi:10.3390/vaccines11020264)
Supplement: Supplementary file 1 [file vaccines-11-00264-s001.zip › Supplementary Table S1.pdf]

Table S1: Mean ± Standard error (SE) and confidence Interval (CI) of different cytokines/chemokines at different time points

TIME POINTS (DAY)	IL-9		IL-15		IL-17A/F		IL-17F	
	MEAN ± SE	CL (95%)	MEAN ± SE	CL (95%)	MEAN ± SE	CL (95%)	MEAN ± SE	CL (95%)
CONTROL GROUP								
0	0.063 ± 0.012;	0.026, 0.100	7.358 ± 0.266;	6.510, 8.206	1.646 ± 0.553;	-0.114, 3.406	77.28 ± 10.78;	43.09, 111.5
0.25	0.066 ± 0.006;	0.046, 0.086	8.145 ± 0.825;	5.519, 10.77	1.496 ± 0.348;	0.387, 2.605	99.17 ± 9.385;	58.80, 139.5
1	0.054 ± 0.010;	0.022, 0.086	8.377 ± 0.954;	5.341, 11.41	1.692 ± 0.587;	-0.176, 3.560	75.54 ± 10.20;	31.67, 119.4
2	0.064 ± 0.007;	0.040, 0.087	8.922 ± 0.659;	6.825, 11.02	1.765 ± 0.480;	0.238, 3.292	83.21 ± 7.211;	60.26, 106.2
3	0.056 ± 0.009;	0.026, 0.086	9.233 ± 0.853;	6.518, 11.95	1.536 ± 0.462;	0.066, 3.006	80.54 ± 12.08;	42.09, 119.0
4	0.052 ± 0.004;	0.041, 0.063	9.141 ± 0.781;	6.657, 11.63	1.778 ± 0.694;	-0.430, 3.986	106.0 ± 8.915;	77.62, 134.4
5	0.040 ± 0.005;	0.025, 0.055	8.919 ± 0.932;	5.952, 11.89	1.354 ± 0.521;	-0.304, 3.012	96.19 ± 10.67;	62.23, 130.2
7	0.054 ± 0.007;	0.031, 0.077	9.513 ± 0.964;	6.447, 12.58	1.537 ± 0.597;	-0.362, 3.437	89.48 ± 5.562;	71.78, 107.2
14	0.049 ± 0.006;	0.031, 0.067	9.620 ± 1.418;	5.107, 14.13	1.305 ± 0.465;	-0.175, 2.785	92.38 ± 10.58;	58.72, 126.0
21	0.053 ± 0.012;	0.015, 0.092	14.46 ± 1.864;	4.523, 16.39	1.229 ± 0.500;	-0.363, 2.822	125.8 ± 16.71;	72.65, 179.0
SIV-INFECTED GROUP								
0	0.047 ± 0.009;	0.026, 0.068	9.314 ± 0.702;	7.727, 10.90	3.264 ± 0.985;	1.037, 5.492	33.73 ± 11.23;	8.321, 56.13
0.25	0.037 ± 0.007;	0.020, 0.054	9.333 ± 0.741;	7.656, 11.01	3.351 ± 0.896;	1.324, 5.379	39.98 ± 9.173;	19.23, 60.74
1	0.010 ± 0.004;	0.001, 0.019	9.830 ± 1.018;	7.527, 12.13	1.313 ± 0.645;	-0.147, 2.772	53.80 ± 14.45;	21.10, 86.49
2	0.009 ± 0.005;	-0.002, 0.020	9.953 ± 0.962;	7.775, 12.13	1.361 ± 0.597;	0.010, 2.712	58.26 ± 12.03;	31.04, 85.48
3	0.011 ± 0.005;	0.000, 0.022	10.18 ± 1.029;	7.852, 12.51	1.081 ± 0.528;	-0.113, 2.275	20.28 ± 6.613;	5.036, 35.53
4	0.017 ± 0.006;	0.003, 0.030	12.41 ± 1.371;	9.310, 15.51	1.017 ± 0.498;	-0.111, 2.144	76.09 ± 12.57;	47.66, 104.5
5	0.020 ± 0.006;	0.006, 0.034	17.45 ± 2.205;	12.46, 22.44	0.840 ± 0.347;	0.054, 1.626	85.82 ± 16.28;	48.99, 122.6
7	0.032 ± 0.009;	0.010, 0.054	23.61 ± 2.517;	17.92, 29.30	2.527 ± 0.603;	1.163, 3.890	151.1 ± 19.30;	107.5, 194.8
14	0.017 ± 0.006;	0.002, 0.031	11.67 ± 0.846;	9.761, 13.59	1.876 ± 0.309;	1.177, 2.575	52.69 ± 12.81;	23.71, 81.68
21	0.036 ± 0.007;	0.021, 0.052	9.838 ± 1.216;	7.087, 12.59	1.761 ± 0.440;	0.467, 2.755	89.73 ± 20.63;	43.06, 136.4

CONTROL GROUP								
TIME POINTS (DAY)	IL-18		IL-1RA		TNF-B		YKL-40	
	MEAN ± SE	CL (95%)	MEAN ± SE	CL (95%)	MEAN ± SE	CL (95%)	MEAN ± SE (10^5)	CL (95%, 10^5)
0	31.25 ± 4.641;	16.48, 46.02	209.5 ± 56.56;	29.54, 389.5	0.044 ± 0.020;	-0.019, 0.107	2.6 ± 0.5;	1.1, 4.2
0.25	31.14 ± 3.580;	19.75, 42.54	240.9 ± 38.75;	117.6, 364.3	0.034 ± 0.018;	-0.022, 0.091	2.4 ± 0.5;	0.8, 4.0
1	30.76 ± 3.887;	18.39, 43.13	171.2 ± 41.19;	40.13, 302.3	0.024 ± 0.016;	-0.026, 0.075	2.9 ± 0.4;	1.7, 4.1
2	32.27 ± 3.187;	22.13, 42.41	157.7 ± 38.07;	36.59, 278.9	0.014 ± 0.009;	-0.016, 0.043	3.4 ± 0.4;	2.3, 4.6
3	30.08 ± 3.291;	19.60, 40.55	160.5 ± 38.56;	37.84, 283.2	0.029 ± 0.014;	-0.015, 0.074	3.5 ± 0.3;	2.5, 4.4
4	30.20 ± 3.832;	18.00, 42.39	206.2 ± 52.10;	40.39, 372.0	0.023 ± 0.023;	-0.050, 0.095	3.4 ± 0.4;	2.3, 4.6
5	31.85 ± 4.452;	17.68, 46.01	239.4 ± 61.96;	42.24, 436.6	0.018 ± 0.014;	-0.026, 0.064	3.5 ± 0.6;	1.5, 5.5
7	33.46 ± 5.884;	14.74, 52.19	171.4 ± 44.24;	30.62, 312.2	0.019 ± 0.014;	-0.026, 0.064	3.8 ± 0.8;	1.0, 6.5
14	32.28 ± 4.241;	18.79, 45.78	165.2 ± 45.41;	20.71, 309.8	0.021 ± 0.013;	-0.020, 0.062	2.7 ± 0.6;	0.7, 4.8
21	38.90 ± 8.025;	13.37, 64.44	230.4 ± 79.39;	-22.24, 483.1	0.128 ± 0.108;	-0.215, 0.471	3.6 ± 1.0;	0.3, 6.9
SIV-INFECTED GROUP								
0	87.89 ± 8.148;	69.46, 106.3	211.5 ± 19.32;	167.8, 255.2	0.119 ± 0.037;	0.034, 0.204	5.6 ± 0.7;	4.0, 7.1
0.25	89.12 ± 9.009;	68.73, 109.5	558.7 ± 231.4;	35.23, 1082	0.100 ± 0.031;	0.029, 0.171	4.3 ± 0.6;	3.0, 5.6
1	60.46 ± 5.729;	47.50, 73.42	225.7 ± 28.69;	160.8, 290.6	0.045 ± 0.029;	-0.021, 0.112	5.0 ± 0.8;	3.2, 6.9
2	65.98 ± 6.549;	51.16, 80.79	219.6 ± 19.37;	175.7, 263.4	0.061 ± 0.030;	-0.008, 0.129	4.8 ± 0.6;	3.4, 6.2
3	66.35 ± 7.581;	49.20, 83.50	247.7 ± 43.84;	148.5, 346.9	0.068 ± 0.036;	-0.012, 0.149	4.8 ± 0.6;	3.3, 6.2
4	68.27 ± 7.101;	52.21, 84.33	454.5 ± 95.75;	237.9, 671.1	0.038 ± 0.019;	-0.004, 0.081	5.7 ± 0.5;	4.5, 7.0
5	68.80 ± 6.505;	54.08, 83.51	804.2 ± 151.2;	462.3, 1146	0.040 ± 0.021;	-0.009, 0.088	6.2 ± 1.2;	3.4, 9.0
7	117.4 ± 13.63;	86.59, 148.3	1236 ± 200.7;	355.4, 1690	0.112 ± 0.024;	0.058, 0.165	4.2 ± 0.7;	2.6, 5.8
14	378.4 ± 74.98;	208.8, 548.0	626.0 ± 119.6;	355.4, 896.6	0.101 ± 0.014;	0.070, 0.131	5.6 ± 1.0;	3.4, 7.8
21	212.9 ± 28.63;	148.1, 277.6	664.9 ± 144.2;	338.6, 991.1	0.111 ± 0.019;	0.068, 0.154	6.3 ± 0.9;	4.2, 8.4

CONTROL GROUP								
TIME POINTS (DAY)	EOTAXIN		FLT3L		IP-10		M-CSF	
	MEAN ± SE	CL (95%)	MEAN ± SE	CL (95%)	MEAN ± SE	CL (95%)	MEAN ± SE	CL (95%)
0	1520 ± 232.8;	779.1, 2261	8.324 ± 2.300;	1.004, 15.64	517.6 ± 105.4;	182.2, 853.1	5.719 ± 1.118;	2.162, 9.277
0.25	1308 ± 159.5;	800.5, 1816	5.100 ± 1.478;	0.397, 9.804	371.3 ± 89.61;	86.09, 656.4	6.185 ± 0.833;	3.533, 8.837
1	1553 ± 246.7;	767.7, 2338	7.376 ± 2.251;	0.211, 14.54	425.0 ± 84.28;	156.8, 693.2	6.623 ± 1.246;	2.657, 10.59
2	1416 ± 192.6;	802.8, 2028	6.998 ± 2.306;	-0.342, 14.34	338.7 ± 80.00;	84.15, 593.3	5.212 ± 0.793;	2.689, 7.736
3	1358 ± 148.4;	885.7, 1830	6.692 ± 2.391;	-0.915, 14.30	399.7 ± 69.12;	179.8, 619.7	5.421 ± 0.997;	2.248, 8.595
4	1175 ± 85.77;	902.1, 1448	5.846 ± 2.000;	-0.518, 12.21	448.5 ± 105.5;	112.9, 784.1	7.816 ± 1.834;	1.979, 13.65
5	2151 ± 374.3;	959.8, 3342	8.425 ± 2.777;	-0.414, 17.26	677.4 ± 148.4;	205.3, 1150	6.274 ± 1.154;	2.602, 9.947
7	1577 ± 115.2;	1210, 1943	8.205 ± 1.965;	1.950, 14.46	459.6 ± 83.92;	192.5, 726.6	5.869 ± 1.163;	2.167, 9.571
14	1756 ± 148.5;	1283, 2228	9.752 ± 2.592;	1.502, 18.00	500.0 ± 59.73;	309.9, 690.0	6.011 ± 0.793;	3.487, 8.535
21	1864 ± 343.9;	769.8, 2959	10.21 ± 3.050;	0.508, 19.92	589.5 ± 132.7;	167.1, 1012	60.50 ± 1.222;	2.162, 9.938
SIV-INFECTED GROUP								
0	2145 ± 357.9;	1335, 2954	16.99 ± 1.846;	12.81, 21.17	570.5 ± 61.36;	431.7, 709.3	6.100 ± 1.075;	3.669, 8.531
0.25	1915 ± 345.8;	1132, 2697	11.75 ± 0.978;	9.542, 13.96	340.5 ± 30.30;	272.0, 409.1	6.972 ± 1.149;	4.374, 9.570
1	1659 ± 313.5;	949.8, 2368	6.426 ± 0.779;	4.665, 8.188	382.9 ± 36.13;	301.1, 464.6	8.760 ± 2.104;	4.001, 13.52
2	1391 ± 214.4;	906.4, 1877	8.095 ± 1.463;	4.785, 11.41	405.7 ± 52.52;	286.8, 524.5	8.057 ± 1.540;	4.574, 11.54
3	1385 ± 188.2;	959.2, 1811	8.486 ± 1.626;	4.806, 12.17	465.1 ± 61.92;	325.0, 605.2	6.786 ± 1.509;	3.371, 10.20
4	1751 ± 372.6;	907.8, 2593	11.36 ± 1.785;	7.324, 15.40	939.3 ± 160.3;	576.6, 1302	8.256 ± 1.521;	4.814, 11.70
5	2173 ± 423.0;	1216, 3130	14.43 ± 2.540;	8.685, 20.18	1761 ± 413.1	826.6, 2696	12.81 ± 2.694;	6.721, 18.91
7	3830 ± 436.5;	2842, 4817	33.88± 4.642	23.37, 44.38	3832 ± 420.3;	2881, 4782	13.72 ± 1.741;	9.784, 17.66
14	2062 ± 303.1;	1376, 2748	53.04 ± 8.178;	34.54, 71.54	1734 ± 312.5;	1027, 2441	12.05 ± 2.932;	5.422, 18.69
21	2613 ± 396.4;	1716, 3510	33.29 ± 7.085;	17.27, 49.32	2485 ± 291.7;	1825, 3145	13.45 ± 4.047;	4.300, 22.61

CONTROL GROUP								
TIME POINTS (DAY)	MCP-1		MCP-2		MCP-4		MDC	
	MEAN ± SE	CL (95%)	MEAN ± SE	CL (95%)	MEAN ± SE	CL (95%)	MEAN ± SE	CL (95%)
0	70.25 ± 3.277;	59.82, 80.68	3.595 ± 0.664;	1.480, 5.710	156.6 ± 33.06;	51.42, 261.8	134.4 ± 13.69;	90.88, 178.0
0.25	76.78 ± 6.122;	57.29, 96.26	2.628 ± 0.449;	1.200, 4.056	111.6 ± 29.99;	16.19, 207.1	137.2 ± 17.11;	82.72, 191.6
1	82.98 ± 8.527;	55.85, 110.1	3.304 ± 0.561;	1.517, 5.019	126.5 ± 41.95;	-7.015, 260.0	137.3 ± 13.53;	94.25, 180.3
2	72.72 ± 7.159;	49.94, 95.51	2.554 ± 0.468;	1.064, 4.044	98.01 ± 23.53;	23.12, 172.9	121.3 ± 13.29;	78.97, 163.6
3	75.41 ± 7.105;	52.80, 98.02	2.846 ± 0.658;	0.752, 4.941	103.2 ± 27.54;	15.52, 190.8	119.6 ± 16.29;	67.81, 171.5
4	71.81 ± 8.098;	46.04, 97.58	2.015 ± 0.534;	0.314, 3.716	115.5 ± 35.00;	4.105, 226.9	109.0 ± 14.19;	63.80, 154.1
5	104.1 ± 14.15;	59.04, 149.1	3.387 ± 0.454;	1.942, 4.832	222.8 ± 53.81;	51.57, 394.0	108.6 ± 11.67;	71.44, 145.7
7	74.00 ± 7.549;	49.98, 98.03	2.794 ± 0.543;	1.066, 4.521	172.7 ± 43.58;	33.97, 311.4	126.9 ± 13.01;	85.51, 168.3
14	65.50 ± 5.894;	46.74, 84.25	3.172 ± 0.513;	1.540, 4.805	183.6 ± 29.84;	88.69, 278.6	139.1 ± 7.285;	116.0, 162.3
21	69.94 ± 8.881;	41.67, 98.20	3.345 ± 0.583;	1.489, 5.201	186.6 ± 28.79;	94.97, 278.2	166.3 ± 13.08;	124.7, 207.9
SIV-INFECTED GROUP								
0	81.39 ± 4.954;	70.18, 92.59	2.700 ± 0.634;	1.266, 4.134	481.8 ± 46.42;	376.7, 586.8	340.6 ± 56.45;	212.9, 468.3
0.25	103.5 ± 13.85;	72.13, 134.8	2.457 ± 0.587;	1.130, 3.785	261.8 ± 27.20;	200.3, 323.4	303.6 ± 49.64;	191.3, 415.9
1	78.20 ± 6.498;	63.50, 92.90	2.064 ± 0.668;	0.554, 3.574	239.7 ± 33.87;	163.1, 316.3	194.6 ± 31.45;	123.4, 265.7
2	84.75 ± 8.809;	64.82, 104.7	2.387 ± 0.673;	0.866, 3.909	218.3 ± 26.34;	158.7, 277.9	149.7 ± 18.90;	107.0, 192.5
3	85.21 ± 8.018;	67.07, 103.3	3.104 ± 0.829;	1.229, 4.978	193.0 ± 26.61;	132.8, 253.2	148.1 ± 20.72;	101.2, 195.0
4	114.9 ± 19.06;	71.76, 158.0	9.748 ± 2.756;	3.512, 15.98	300.3 ± 44.89;	198.8, 401.9	147.2 ± 17.99;	106.5, 187.9
5	156.1 ± 19.97;	111.0, 201.3	24.97 ± 7.160;	8.774, 41.17	328.8 ± 43.17;	231.1, 426.5	166.8 ± 21.92;	117.2, 216.4
7	249.4 ± 30.93;	179.4, 319.4	56.07± 11.65;	29.71, 82.43	600.9 ± 73.83;	433.9, 767.9	256.6 ± 39.83;	166.5, 346.8
14	137.1 ± 10.80;	112.7, 161.5	6.552 ± 0.986;	4.320, 8.783	321.8 ± 54.74;	197.9, 445.6	250.8 ± 39.83;	160.7, 340.9
21	119.2 ± 6.172;	105.2, 133.2	7.179 ± 1.150;	4.578, 9.780	387.6 ± 60.22;	251.4, 523.8	382.6 ± 66.29;	232.6, 532.6

CONTROL GROUP								
TIME POINTS (DAY)	MIP-1A		MIP-3B		SDF-1A		VEGF	
	MEAN ± SE	CL (95%)	MEAN ± SE	CL (95%)	MEAN ± SE	CL (95%)	MEAN ± SE	CL (95%)
0	15.76 ± 1.193;	11.96, 19.55	29.44 ± 2.254;	22.26, 36.61	2475 ± 1638;	-2736, 7687	2.600 ± 0.738;	0.251, 4.950
0.25	18.67 ± 4.590;	4.059, 33.27	28.59 ± 3.005;	19.03, 38.16	950.8 ± 72.41;	720.3, 1181	3.755 ± 0.722;	1.459, 6.051
1	17.91 ± 5.531;	0.308, 35.51	30.24 ± 3.358;	19.56, 40.93	874.8 ± 72.43;	644.3, 1105	3.248 ± 0.374;	2.057, 4.440
2	11.57 ± 0.673;	9.427, 13.71	28.74 ± 3.019;	19.14, 38.35	910.8 ± 26.12;	827.7, 994.0	3.819 ± 0.681;	1.652, 5.986
3	12.74 ± 0.509;	11.12, 14.36	30.53 ± 4.244;	17.02, 44.03	889.8 ± 47.05;	740.1, 1040	3.318 ± 0.451;	1.883, 4.754
4	13.23 ± 0.903;	10.35, 16.10	30.75 ± 4.879;	15.22, 46.27	848.8 ± 79.84;	594.7, 1103	3.662 ± 0.730;	1.340, 5.983
5	13.85 ± 0.852;	11.14, 16.56	33.01 ± 3.772;	21.00, 45.01	882.0 ± 67.98;	665.7, 1098	4.754 ± 0.624;	2.769, 6.738
7	12.65 ± 0.633;	10.64, 14.67	34.03 ± 5.542;	16.40, 51.67	1039 ± 73.75	804.5, 1274	3.716 ± 0.669;	1.586, 5.846
14	14.84 ± 2.382;	7.259, 22.42	37.23 ± 2.620;	28.89, 45.57	897.9 ± 48.99;	742.0, 1054	2.630 ± 0.558;	0.853, 4.406
21	15.44 ± 1.950;	9.232, 21.64	33.19 ± 3.701;	21.42, 44.97	1766 ± 732.4	-564.7, 4097	3.386 ± 0.725;	1.079, 5.693
SIV-INFECTED GROUP								
0	28.30 ± 4.400;	18.35, 38.26	46.30 ± 5.977;	32.78, 59.82	2734 ± 644.9;	1275, 4193	6.426 ± 0.527;	5.235, 7.618
0.25	22.26 ± 1.954;	17.84, 26.68	38.98 ± 2.766;	32.72, 45.23	2503 ± 588.6;	1172, 3835	7.539 ± 0.850;	5.617, 9.461
1	20.23 ± 5.086;	8.720, 31.73	37.49 ± 1.777;	33.47, 41.51	1720 ± 535.8;	507.7, 2932	5.236 ± 0.530;	4.039, 6.434
2	20.48 ± 3.480;	12.61, 28.35	33.63 ± 1.860;	29.43, 37.84	1895 ± 596.3;	546.1, 3244	6.838 ± 0.648;	5.372, 8.305
3	19.23 ± 3.008;	12.43, 26.04	40.74 ± 3.799;	32.14, 49.33	1871 ± 602.6;	507.7, 3234	5.591 ± 0.517;	4.782, 7.120
4	22.00 ± 2.942;	15.34, 28.65	38.44 ± 2.549;	32.67, 44.20	1931 ± 543.2;	701.8, 3160	7.871 ± 1.489;	4.502, 11.24
5	26.98 ± 3.038;	20.10, 33.85	49.70 ± 5.058;	38.25, 61.14	1911 ± 528.3;	715.9, 3106	7.218 ± 0.829;	5.342, 9.094
7	38.81 ± 3.278;	31.39, 46.23	78.44± 8.437;	59.36, 97.53	2834 ± 630.8;	1407, 4261	7.708 ± 0.744;	6.025, 9.391
14	20.77 ± 1.905;	16.46, 25.07	151.0 ± 22.37;	100.4, 201.6	2316 ± 578.3;	1007, 3624	6.479 ± 0.514;	5.263, 7.694
21	33.09 ± 9.315;	12.02, 54.16	226.2 ± 32.53;	152.6, 299.8	3178 ± 713.2;	1564, 4791	6.276 ± 1.003;	4.008, 8.544

CONTROL GROUP								
TIME POINTS (DAY)	CTACK		ENA-78		FRACTALKINE		IL-13	
	MEAN ± SE	CL (95%)	MEAN ± SE	CL (95%)	MEAN ± SE	CL (95%)	MEAN ± SE	CL (95%)
0	1979 ± 399.2;	708.2, 3249	148.8 ± 16.84;	95.23, 202.4	12300 ± 1155;	8624, 15975	12.22 ± 1.835;	6.377, 18.06
0.25	1775 ± 329.8;	725.4, 2825	125.1 ± 19.79;	62.14, 188.1	13386 ± 1170;	9662, 17110	12.77 ± 2.364;	5.249, 20.30
1	1835 ± 374.9;	642.5, 3028	130.8 ± 21.44;	62.53, 199.0	14096 ± 1438;	9519, 18672	13.26 ± 4.206;	-0.125, 26.65
2	1951 ± 408.4;	650.7, 3250	116.9 ± 14.07;	72.15, 161.7	12843 ± 1271;	8800, 16887	12.33 ± 1.735;	6.803, 17.85
3	1946 ± 353.6;	821.0, 3072	122.5 ± 21.77;	53.19, 191.8	13499 ± 1471;	8817, 18182	8.761 ± 1.390;	4.339, 13.18
4	1799 ± 347.0;	694.2, 2903	100.3 ± 15.36;	51.39, 149.1	11386 ± 1249;	7411, 15362	9.110 ± 2.404;	1.461, 16.76
5	2173 ± 370.1;	995.6, 3351	121.8 ± 18.28;	63.66, 180.0	11864 ± 982.2;	8738, 14989	7.958 ± 2.087;	1.318, 14.60
7	2018 ± 363.9;	860.2, 3177	111.3 ± 16.64;	58.35, 164.3	12003 ± 934.1	9031, 14976	9.393 ± 1.882;	3.405, 15.38
14	1935 ± 391.9;	687.9, 3182	95.23 ± 13.43;	52.50, 138.0	9762 ± 630.1	7757, 11767	4.057 ± 1.512;	-0.754, 8.867
21	1885 ± 480.1;	352.7, 3413	116.0 ± 18.94;	55.79, 176.3	10747 ± 1347	6461, 15032	12.00 ± 1.647;	6.761, 17.24
SIV-INFECTED GROUP								
0	1829 ± 213.2;	1347, 2311	59.36 ± 6.193;	45.35, 73.37	9190 ± 684.3;	7723, 10656	4.252 ± 2.366;	-1.100, 9.604
0.25	1781 ± 179.3;	1376, 2187	49.38 ± 4.280;	39.70, 59.06	10382 ± 832.7;	8499, 12266	5.337 ± 1.890;	1.061, 9.612
1	1518 ± 126.0;	1233, 1803	41.00 ± 6.379;	26.57, 55.43	7507 ± 662.9;	6008, 9007	1.843 ± 1.588;	-1.749, 5.434
2	1594 ± 114.4;	1335, 1853	36.36 ± 6.789;	21.00, 51.72	7924 ± 713.2;	6311, 9538	1.591 ± 1.591;	-2.008, 5.189
3	1610 ± 130.0;	1316, 1904	36.87 ± 4.763;	26.10, 47.64	7725 ± 673.7;	6201, 9249	2.181 ± 1.557;	-1.341, 5.703
4	1608 ± 108.6;	1362, 1853	34.47 ± 3.846;	25.77, 43.17	8093 ± 665.4;	6588, 9598	0.980 ± 0.934;	-1.131, 3.092
5	1508 ± 133.9;	1205, 1811	40.00 ± 3.884;	31.21, 48.78	7909 ± 609.5;	6530, 9288	0.937 ± 0.937;	-1.183, 3.057
7	1905 ± 184.4;	1487, 2322	85.82 ± 5.590	73.17, 98.46	10215 ± 806.4;	8391, 12039	7.386 ± 1.689;	3.566, 11.21
14	1921 ± 185.5;	1501, 2340	83.98 ± 9.765;	61.89, 106.1	15017 ± 1402;	11846, 18188	6.421 ± 1.362;	3.340, 9.503
21	2173 ± 226.2;	1661, 2685	78.77 ± 6.579;	63.89, 93.65	10488 ± 1466;	7171, 13805	4.387 ± 1.469;	1.063, 7.710

CONTROL GROUP								
TIME POINTS (DAY)	IL-17A		I-TAC		MIP-3A		TNF-A	
	MEAN ± SE	CL (95%)	MEAN ± SE	CL (95%)	MEAN ± SE	CL (95%)	MEAN ± SE	CL (95%)
0	0.740 ± 0.207;	-0.120, 1.601	108.6 ± 34.22;	-0.336, 217.5	3.629 ± 0.542;	1.903, 5.355	0.482 ± 0.106;	0.146, 0.819
0.25	1.165 ± 0.476;	-0.349, 2.678	88.41 ± 27.07;	2.253, 174.6	3.270 ± 0.534;	1.571, 4.970	0.508 ± 0.117;	0.135, 0.880
1	1.163 ± 0.950;	-1.861, 4.186	111.0 ± 44.97;	-32.14, 254.1	3.526 ± 0.821;	0.913, 6.140	0.415 ± 0.071;	0.188, 0.642
2	0.943 ± 0.318;	-0.070, 1.956	93.26 ± 34.61;	-16.88, 203.4	4.171 ± 1.065;	0.782, 7.559	0.415 ± 0.053;	0.245, 0.584
3	0.275 ± 0.154;	-0.215, 0.765	115.9 ± 33.12;	10.47, 221.3	3.118 ± 0.522;	1.456, 4.780	0.307 ± 0.079;	0.056, 0.558
4	0.343 ± 0.272;	-0.522, 1.208	128.4 ± 45.50;	-16.37, 273.2	3.596 ± 0.649;	1.531, 5.661	0.253 ± 0.072;	0.022, 0.483
5	0.263 ± 0.162;	-0.252, 0.777	148.1 ± 54.23;	-24.49, 320.7	4.025 ± 0.520;	2.369, 5.681	0.342 ± 0.087;	0.064, 0.620
7	0.550 ± 0.449;	-0.880, 1.980	153.2 ± 75.95;	-88.54, 394.9	3.638 ± 0.350;	2.254, 4.752	0.369 ± 0.103;	0.041, 0.697
14	0.134 ± 0.116;	-0.234, 0.503	145.1 ± 31.57;	44.67, 245.6	4.122 ± 0.602;	2.208, 6.037	0.253 ± 0.054;	0.080, 0.426
21	0.805 ± 0.487;	-0.746, 2.356	208.2 ± 102.7;	-118.7, 535.2	3.901± 0.714;	1.627, 6.174	0.481 ± 0.124;	0.086, 0.875
SIV-INFECTED GROUP								
0	0.313 ± 0.218;	-0.180, 0.806	148.6 ± 19.79;	103.9, 193.4	4.720 ± 0.335;	3.963, 5.476	0.228 ± 0.068;	0.074, 0.382
0.25	0.263 ± 0.171;	-0.123, 0.650	127.0 ± 14.86;	93.34, 160.6	4.526 ± 0.262;	3.935, 5.118	0.189 ± 0.068;	0.035, 0.343
1	0.032 ± 0.032;	-0.041, 0.105	125.1 ± 15.99;	88.97, 161.3	4.554 ± 0.441;	3.556, 5.551	0.093 ± 0.035;	0.013, 0.173
2	0.039 ± 0.039;	-0.050, 0.129	154.0 ± 27.16;	92.56, 215.5	4.274 ± 0.242;	3.726, 4.821	0.039 ± 0.024;	-0.015, 0.092
3	0.044 ± 0.044;	-0.055, 0.142	148.5 ± 18.22;	107.3, 189.7	4.232 ± 0.338;	3.468, 4.996	0.024 ± 0.015;	-0.010, 0.058
4	0.029 ± 0.029;	-0.037, 0.096	236.0 ± 37.14;	152.0, 320.0	4.155 ± 0.455;	3.126, 5.185	0.004 ± 0.004;	-0.005, 0.012
5	0.000 ± 0.000;	0.000, 0.000	532.2 ± 108.9;	285.9, 778.4	4.726 ± 0.550;	3.483, 5.969	0.001 ± 0.001;	-0.001, 0.002
7	0.398 ± 0.179;	-0.006, 0.803	973.3 ± 131.7;	675.4, 1271	6.017 ± 0.558;	4.756, 7.279	0.147 ± 0.043;	0.049, 0.244
14	0.456 ± 0.185;	-0.039, 0.874	740.6 ± 113.9;	482.9, 998.2	4.263 ± 0.270;	3.651, 4.874	0.216 ± 0.064;	0.070, 0.362
21	0.248 ± 0.141;	-0.072, 0.567	902.8 ± 131.9;	604.5, 1201	4.975 ± 0.408;	4.052, 5.898	0.079 ± 0.044;	-0.021, 0.179

CONTROL GROUP								
TIME POINTS (DAY)	EOTAXIN-3		MIP-1B		TARC		G-CSF	
	MEAN ± SE	CL (95%)	MEAN ± SE (10^6)	CL (95%, 10^6)	MEAN ± SE	CL (95%)	MEAN ± SE	CL (95%)
0	10.36 ± 2.106;	3.652, 17.06	2.9 ± 0.2;	2.1, 3.7	0.168 ± 0.018;	0.109, 0.226	43.85 ± 11.75;	6.445, 81.25
0.25	8.521 ± 0.867;	5.762, 11.28	2.8 ± 0.2;	2.2, 3.4	0.090 ± 0.016;	0.038, 0.143	25.40 ± 7.484;	1.585, 49.22
1	8.172 ± 1.104;	4.660, 11.68	2.7 ± 0.1;	2.3, 3.1	0.105 ± 0.020;	0.039, 0.170	58.71 ± 8.096;	32.94, 84.47
2	9.095 ± 1.100;	5.593, 12.60	2.9 ± 0.3;	2.1, 3.8	0.104 ± 0.016;	0.052, 0.156	38.24 ± 12.31;	-0.946, 77.42
3	6.990 ± 0.798;	4.450, 9.531	3.3 ± 0.2;	2.7, 3.8	0.104 ± 0.019;	0.045, 0.164	41.67 ± 14.14;	-3.336, 86.68
4	7.887 ± 0.945;	4.878, 10.90	3.5 ± 0.4;	2.3, 4.8	0.106 ± 0.016;	0.054, 0.158	29.12 ± 9.029;	0.388, 57.86
5	9.372 ± 1.171;	5.646, 13.10	3.4 ± 0.4;	2.0, 4.7	0.114 ± 0.016;	0.062, 0.165	29.99 ± 9.091;	1.058, 58.92
7	8.321 ± 0.719;	6.032, 10.61	3.4 ± 0.2;	2.7, 4.2	0.104 ± 0.013;	0.062, 0.146	36.39 ± 21.73;	-32.75, 105.5
14	8.536 ± 0.630;	6.532, 10.54	3.8 ± 0.4;	2.4, 5.3	0.076 ± 0.010;	0.043, 0.110	51.60 ± 19.67;	-10.98, 114.2
21	11.64 ± 1.709;	6.200, 17.08	4.8 ± 1.0;	1.4, 8.2	0.203 ± 0.029;	0.110, 0.296	32.44 ± 13.38;	-10.12, 75.01
SIV-INFECTED GROUP								
0	4.015 ± 0.914;	1.948, 6.082	5.2 ± 0.6;	3.9, 6.5	0.125 ± 0.034;	0.049, 0.201	2.135 ± 0.714;	0.519, 3.751
0.25	2.218 ± 0.588;	0.887, 3.548	5.4 ± 0.6;	4.0, 6.8	0.088 ± 0.035;	0.010, 0.167	1.408 ± 0.374;	0.563, 2.253
1	5.693 ± 1.142;	3.110, 8.277	3.8 ± 0.5;	2.6, 4.9	0.169 ± 0.047;	0.063, 0.275	0.798 ± 0.398;	-0.103, 1.699
2	4.182 ± 1.159;	1.560, 6.804	3.9 ± 0.4;	2.9, 4.8	0.140 ± 0.039;	0.053, 0.228	0.887 ± 0.390;	0.006, 1.769
3	4.898 ± 1.246;	2.078, 7.717	4.1 ± 0.6;	2.7, 5.5	0.183 ± 0.046;	0.080, 0.287	1.876 ± 1.154;	-0.735, 4.487
4	5.493 ± 1.295;	2.563, 8.422	4.7 ± 0.7;	3.1, 6.4	0.551 ± 0.310;	-0.150, 1.251	0.411 ± 0.227;	-0.102, 0.923
5	8.969 ± 1.772;	4.960, 12.98	5.2 ± 0.6;	3.9, 6.4	0.269 ± 0.068;	0.116, 0.422	4.471 ± 3.509;	-3.465, 12.41
7	7.853 ± 1.204;	5.128, 10.58	8.4 ± 1.2;	5.5, 11.2	0.202 ± 0.032;	0.130, 0.274	2.689 ± 0.825;	0.823, 4.556
14	9.601 ± 5.968;	-3.899, 23.10	4.2 ± 0.5;	3.2, 5.3	0.092 ± 0.019;	0.050, 0.135	5.597 ± 1.835;	1.445, 9.749
21	4.849 ± 0.799;	3.041, 6.657	5.0 ± 0.7;	3.4, 6.7	0.172 ± 0.014;	0.141, 0.202	3.577 ± 1.073;	1.151, 6.004

CONTROL GROUP								
TIME POINTS (DAY)	IFN-A2A		IL-12		IL-16		IL-1A	
	MEAN ± SE	CL (95%)	MEAN ± SE	CL (95%)	MEAN ± SE	CL (95%)	MEAN ± SE	CL (95%)
0	0.000 ± 0.000;	0.000, 0.000	58.00 ± 11.60;	21.08, 94.92	113.9 ± 19.55;	51.67, 176.1	0.498 ± 0.129;	0.087, 0.908
0.25	0.000 ± 0.000;	0.000, 0.000	58.01 ± 11.77;	20.54, 95.47	128.2 ± 17.86;	71.34, 185.0	0.286 ± 0.072;	0.057, 0.514
1	0.000 ± 0.000;	0.000, 0.000	54.70 ± 10.35;	21.75, 87.65	98.27 ± 23.76;	22.67, 173.9	0.504 ± 0.237;	-0.249, 1.257
2	0.000 ± 0.000;	0.000, 0.000	59.23 ± 11.58;	22.37, 96.09	79.41 ± 11.48;	42.88, 115.9	0.356 ± 0.068;	0.139, 0.573
3	0.000 ± 0.000;	0.000, 0.000	62.11 ± 12.78;	21.44, 102.8	61.09 ± 12.68;	20.74, 101.4	0.464 ± 0.149;	-0.009, 0.938
4	0.000 ± 0.000;	0.000, 0.000	71.57 ± 15.51;	22.22, 120.9	101.5 ± 20.13;	37.42, 165.6	0.829 ± 0.225;	0.112, 1.546
5	0.000 ± 0.000;	0.000, 0.000	60.55 ± 11.82;	22.95, 98.16	133.9 ± 30.90;	35.60, 232.3	0.586 ± 0.198;	-0.042, 1.215
7	0.000 ± 0.000;	0.000, 0.000	53.58 ± 9.492;	23.37, 83.78	79.77 ± 13.10;	38.07, 121.5	0.505 ± 0.166;	-0.025, 1.035
14	0.000 ± 0.000;	0.000, 0.000	63.82 ± 12.88;	22.84, 104.8	76.44 ± 16.39;	24.28, 128.6	0.979 ± 0.278;	0.095, 1.862
21	1.039 ± 1.039;	-2.268, 4.347	78.21 ± 19.50;	16.15, 140.3	92.35 ± 23.24;	18.40, 166.3	0.551 ± 0.171;	0.008, 1.094
SIV-INFECTED GROUP								
0	0.042 ± 0.042;	-0.053, 0.136	99.12 ± 14.40;	66.56, 131.7	99.73 ± 15.21;	65.31, 134.1	0.363 ± 0.160;	0.001, 0.724
0.25	0.000 ± 0.000;	0.000, 0.000	89.37 ± 9.034;	68.93, 109.8	97.04 ± 13.50;	66.49, 127.6	0.214 ± 0.117;	-0.052, 0.479
1	0.000 ± 0.000;	0.000, 0.000	84.82 ± 10.60;	60.84, 108.8	91.42 ± 15.27;	56.88, 125.9	0.024 ± 0.022;	-0.024, 0.073
2	0.007 ± 0.007;	-0.008, 0.022	88.53 ± 11.16;	63.28, 113.8	106.1 ± 20.53;	59.68, 152.5	0.058 ± 0.035;	-0.022, 0.138
3	0.000 ± 0.000;	0.000, 0.000	88.76 ± 12.75;	59.93, 117.6	96.64 ± 26.70;	36.25, 157.0	0.040 ± 0.028;	-0.024, 0.104
4	0.000 ± 0.000;	0.000, 0.000	77.43 ± 10.19;	54.37, 100.5	115.7 ± 38.78;	27.99, 203.4	0.000 ± 0.000;	0.000, 0.000
5	0.000 ± 0.000;	0.000, 0.000	67.71 ± 6.416;	53.20, 82.22	85.30 ± 22.16;	35.17, 135.4	0.015 ± 0.015;	-0.018, 0.049
7	2.527 ± 1.179;	-0.139, 5.193	78.74 ± 8.069;	60.48, 96.99	77.14 ± 10.00;	54.52, 99.76	0.339 ± 0.164;	-0.032, 0.711
14	0.000 ± 0.000;	0.000, 0.000	124.1 ± 12.68;	95.43, 152.8	80.65 ± 14.22;	48.49, 112.8	0.349 ± 0.190;	-0.082, 0.779
21	0.110 ± 0.066;	-0.040, 0.260	213.7 ± 54.68;	89.98, 337.4	113.6 ± 28.85;	48.38, 178.9	0.273 ± 0.090;	0.068, 0.477

CONTROL GROUP								
TIME POINTS (DAY)	IL-7		TPO		EOTAXIN-2		IL-17B	
	MEAN ± SE	CL (95%)	MEAN ± SE	CL (95%)	MEAN ± SE	CL (95%)	MEAN ± SE	CL (95%)
0	3.005 ± 0.851;	0.296, 5.714	137.0 ± 14.33;	91.40, 182.6	27.04 ± 5.993;	7.970, 46.11	0.068 ± 0.014;	0.022, 0.114
0.25	1.928 ± 0.416;	0.603, 3.252	119.0 ± 12.72;	78.54, 159.5	16.73 ± 4.304;	3.031, 30.42	0.100 ± 0.003;	0.089, 0.111
1	1.530 ± 0.344;	0.434, 2.625	124.4 ± 16.82;	70.90, 178.0	16.25 ± 4.112;	3.158, 29.33	0.126 ± 0.027;	0.040, 1.212
2	1.267 ± 0.288;	0.351, 2.182	121.7 ± 13.74;	78.02, 165.5	13.45 ± 2.928;	4.132, 22.77	0.125 ± 0.009;	0.098, 0.154
3	1.442 ± 0.363;	0.287, 2.596	121.7 ± 17.34;	66.48, 176.8	12.74 ± 2.962;	3.319, 22.17	0.106 ± 0.012;	0.067, 0.146
4	1.932 ± 0.458;	0.473, 3.391	129.6 ± 21.07;	62.57, 196.7	16.14 ± 3.349;	5.483, 26.80	0.111 ± 0.006;	0.090, 1.132
5	2.160 ± 0.568;	0.354, 3.965	139.6 ± 19.32;	78.14, 201.1	21.06 ± 2.300;	13.74, 28.38	0.091 ± 0.015;	0.043, 1.139
7	1.324 ± 0.262;	0.492, 2.156	113.7 ± 14.38;	67.97, 159.5	14.16 ± 3.041;	4.489, 23.84	0.123 ± 0.014;	0.077, 1.169
14	1.694 ± 0.362;	0.543, 2.854	125.3 ± 19.91;	61.95, 188.7	21.51 ± 6.583;	0.554, 42.46	0.115 ± 0.017;	0.060, 1.170
21	1.694 ± 0.222;	0.983, 2.402	125.6 ± 20.67;	59.87, 191.4	21.26 ± 7.707;	-3.271, 45.78	0.089 ± 0.020;	0.025, 1.153
SIV-INFECTED GROUP								
0	9.154 ± 1.647;	5.428, 12.88	191.4 ± 18.40;	149.8, 233.0	49.05 ± 10.56;	25.16, 72.94	0.024 ± 0.008;	0.005, 0.043
0.25	4.183 ± 0.564;	2.908, 5.458	148.6 ± 8.372;	129.7, 167.6	22.40 ± 2.538;	16.66, 28.14	0.012 ± 0.007;	-0.004, 0.029
1	4.790 ± 0.855;	2.856, 6.725	149.7 ± 11.17;	124.5, 175.0	20.65 ± 4.470;	10.53, 30.76	0.002 ± 0.001;	-0.002, 0.005
2	8.111 ± 1.389;	4.969, 11.25	186.0 ± 19.16;	142.6, 229.3	29.88 ± 5.398;	17.67, 42.09	0.000 ± 0.000;	0.000, 0.000
3	4.759 ± 0.857;	2.819, 6.698	150.3 ± 13.66;	119.4, 181.2	23.11 ± 4.499;	12.93, 33.28	0.006 ± 0.006;	-0.007, 0.020
4	4.850 ± 0.726;	3.208, 6.491	158.5 ± 13.03;	129.0, 188.0	20.76 ± 4.108;	11.46, 30.05	0.000 ± 0.000;	0.000, 0.000
5	7.569 ± 2.467;	1.985, 13.15	185.9 ± 26.82;	125.2, 246.5	41.75 ± 21.73;	-7.418, 90.92	0.000 ± 0.000;	0.000, 0.000
7	10.69 ± 3.978;	1.696, 19.69	221.1 ± 38.79;	133.4, 308.8	26.01 ± 7.447;	9.160, 42.85	0.006 ± 0.004;	-0.004, 0.016
14	6.631 ± 0.923;	4.543, 8.718	208.5 ± 24.52;	153.0, 263.9	21.31 ± 2.774;	15.03, 27.58	0.038 ± 0.016;	0.000, 0.075
21	6.268 ± 0.913;	4.204, 8.333	204.5 ± 32.86;	130.2, 278.9	29.60 ± 5.300;	17.61, 41.59	0.077 ± 0.024;	0.022, 0.132

CONTROL GROUP								
TIME POINTS (DAY)	IL-17C		IL-17D		IL-22		IL-23	
	MEAN ± SE	CL (95%)	MEAN ± SE	CL (95%)	MEAN ± SE	CL (95%)	MEAN ± SE	CL (95%)
0	0.740 ± 0.327;	-0.300, 1.780	6.500 ± 1.414;	1.999, 11.00	0.012 ± 0.009;	-0.016, 0.040	1.248 ± 0.653;	-0.830, 3.327
0.25	0.907 ± 0.089;	0.625, 1.190	7.443 ± 2.073;	0.846, 14.04	0.007 ± 0.006;	-0.012, 0.027	2.090 ± 1.084;	-1.359, 5.540
1	1.105 ± 0.177;	0.542, 1.669	6.637 ± 2.284;	-0.634, 13.91	0.005 ± 0.003;	-0.005, 0.014	1.061 ± 0.592;	-0.824, 2.945
2	1.070 ± 0.117;	0.698, 1.442	7.328 ± 2.559;	-0.818, 15.47	0.023 ± 0.015;	-0.023, 0.070	0.893 ± 0.496;	-0.684, 2.470
3	0.844 ± 0.057;	0.661, 1.027	8.084 ± 2.138;	1.282, 14.89	0.033 ± 0.025;	-0.047, 0.112	1.270 ± 0.544;	-0.461, 3.000
4	0.952 ± 0.187;	0.356, 1.548	7.789 ± 2.338;	0.347, 15.23	0.033 ± 0.030;	-0.061, 0.127	1.118 ± 0.566;	-0.684, 2.920
5	0.934 ± 0.216;	0.249, 1.620	7.620 ± 2.207;	0.598, 14.64	0.027 ± 0.024;	-0.048, 0.102	1.197 ± 0.553;	-0.564, 2.958
7	0.948 ± 0.161;	0.435, 1.460	7.761 ± 2.095;	1.095, 14.43	0.031 ± 0.022;	-0.038, 0.100	0.897 ± 0.486;	-0.649, 2.443
14	1.022 ± 0.215;	0.336, 1.707	6.643 ± 1.797;	0.923, 12.36	0.008 ± 0.005;	-0.008, 0.024	1.257 ± 0.687;	-0.930, 3.444
21	0.548 ± 0.205;	-0.105, 1.201	7.892 ± 2.328;	0.482, 15.30	0.003 ± 0.003;	-0.008, 0.014	1.473 ± 0.799;	-1.069, 4.015
SIV-INFECTED GROUP								
0	1.396 ± 1.278;	-1.494, 4.286	8.558± 1.654;	4.817, 12.30	0.137 ± 0.041;	0.044, 0.230	0.638 ± 0.276;	0.013, 1.263
0.25	1.223 ± 1.054;	-1.162, 3.608	9.172 ± 1.315;	6.199, 12.15	0.162 ± 0.045;	0.060, 0.263	0.617 ± 0.266;	0.015, 1.219
1	0.890 ± 0.841;	-1.012, 2.791	7.101 ± 1.148;	4.503, 6.698	0.040 ± 0.022;	-0.011, 0.090	0.339 ± 0.178;	-0.064, 0.741
2	0.861 ± 0.822;	-1.000, 2.721	7.143 ± 1.094;	4.667, 9.618	0.025 ± 0.019;	-0.017, 0.067	0.393 ± 0.200;	-0.058, 0.844
3	0.478 ± 0.475;	-0.597, 1.554	6.685 ± 1.553;	3.173, 10.20	0.025 ± 0.014;	-0.007, 0.056	0.244 ± 0.131;	-0.052, 0.539
4	0.387 ± 0.358;	-0.422, 1.196	7.432 ± 1.739;	3.498, 11.37	0.030 ± 0.014;	-0.001, 0.061	0.243 ± 0.123;	-0.035, 0.522
5	0.318 ± 0.266;	-0.284, 0.920	8.003 ± 1.904;	3.697, 12.31	0.020 ± 0.011;	-0.005, 0.046	0.236 ± 0.119;	-0.032, 0.504
7	0.768 ± 0.550;	-0.476, 2.011	8.664 ± 1.499;	5.273, 12.05	0.160 ± 0.029;	0.095, 0.225	0.822 ± 0.358;	0.012, 1.633
14	0.636 ± 0.557;	-0.625, 1.896	8.427 ± 1.464;	5.114, 11.74	0.318 ± 0.217;	-0.174, 0.809	1.619 ± 0.497;	0.495, 2.743
21	0.530 ± 0.348;	-0.258, 1.318	25.44 ± 14.06;	-6.375, 57.25	1.564 ± 1.351;	-1.491, 4.619	1.091 ± 0.449;	0.075, 2.107

CONTROL GROUP								
TIME POINTS (DAY)	IL-2RA		GM-CSF		IFN-G		IL-10	
	MEAN ± SE	CL (95%)	MEAN ± SE	CL (95%)	MEAN ± SE	CL (95%)	MEAN ± SE	CL (95%)
0	5.410 ± 2.477;	-2.472, 13.29	0.000 ± 0.000;	0.000, 0.000	0.341 ± 0.063;	0.141, 0.540	0.020 ± 0.004;	0.006, 0.034
0.25	52.01 ± 46.11;	-94.74, 198.8	0.000 ± 0.000;	0.000, 0.000	0.053 ± 0.032;	-0.049, 0.155	0.008 ± 0.006;	-0.010, 0.026
1	4.320 ± 1.509;	-0.483, 9.123	0.000 ± 0.000;	0.000, 0.000	0.077 ± 0.050;	-0.082, 0.235	0.011 ± 0.006;	-0.007, 0.029
2	4.717 ± 1.787;	-0.970, 10.40	0.000 ± 0.000;	0.000, 0.000	0.065 ± 0.027;	-0.022, 0.152	0.012 ± 0.007;	-0.011, 0.036
3	5.341 ± 2.095;	-1.327, 12.01	0.000 ± 0.000;	0.000, 0.000	0.128 ± 0.074;	-0.108, 0.364	0.013 ± 0.007;	-0.009, 0.035
4	4.628 ± 1.950;	-1.577, 10.83	0.000 ± 0.000;	0.000, 0.000	0.103 ± 0.050;	-0.057, 0.263	0.008 ± 0.004;	-0.004, 0.021
5	4.386 ± 2.143;	-2.436, 11.21	0.000 ± 0.000;	0.000, 0.000	0.129 ± 0.064;	-0.073, 0.331	0.002 ± 0.002;	-0.004, 0.009
7	5.577 ± 2.281;	-1.683, 12.84	0.000 ± 0.000;	0.000, 0.000	0.097 ± 0.042;	-0.038, 0.232	0.014 ± 0.008;	-0.013, 0.040
14	4.974 ± 2.018;	-1.448, 11.40	0.000 ± 0.000;	0.000, 0.000	0.100 ± 0.065;	-0.106, 0.307	0.012 ± 0.004;	-0.001, 0.025
21	2.834 ± 1.636;	-2.373, 8.041	0.000 ± 0.000;	0.000, 0.000	0.065 ± 0.042;	-0.069, 0.199	0.006 ± 0.003;	-0.003, 0.014
SIV-INFECTED GROUP								
0	12.41 ± 8.599;	-7.046, 31.86	0.019 ± 0.019;	-0.024, 0.063	0.545 ± 0.277;	-0.083, 1.172	0.051 ± 0.027;	-0.010, 0.113
0.25	13.08 ± 7.612;	-4.141, 30.30	0.014 ± 0.014;	-0.017, 0.044	0.590 ± 0.264;	-0.007, 1.186	0.048 ± 0.024;	-0.007, 0.102
1	6.275 ± 5.416;	-5.977, 18.53	0.017 ± 0.017;	-0.022, 0.057	0.320 ± 0.175;	-0.075, 0.715	0.031 ± 0.022;	-0.018, 0.080
2	5.322 ± 4.797;	-5.530, 16.17	0.021 ± 0.021;	-0.027, 0.069	0.403 ± 0.250;	-0.162, 0.968	0.029 ± 0.023;	-0.023, 0.081
3	4.847 ± 4.322;	-4.931, 14.62	0.007 ± 0.007;	-0.009, 0.024	0.303 ± 0.152;	-0.040, 0.646	0.026 ± 0.017;	-0.013, 0.065
4	3.971 ± 2.911;	-2.614, 10.56	0.014 ± 0.014;	-0.018, 0.046	0.418 ± 0.203;	-0.039, 0.877	0.030 ± 0.022;	-0.020, 0.081
5	3.474 ± 2.523;	-2.235, 9.182	0.000 ± 0.000;	0.000, 0.000	0.127 ± 0.078;	-0.051, 0.304	0.020 ± 0.012;	-0.008, 0.047
7	7.124 ± 3.911;	-1.722, 15.97	0.000 ± 0.000;	0.000, 0.000	0.370 ± 0.179;	-0.034, 0.775	0.042 ± 0.015;	0.007, 0.076
14	5.433 ± 1.847;	1.256, 9.611	0.000 ± 0.000;	0.000, 0.000	0.326 ± 0.115;	0.066, 0.585	0.034 ± 0.008;	0.017, 0.051
21	2.835 ± 1.459;	-0.465, 6.135	0.000 ± 0.000;	0.000, 0.000	0.423 ± 0.182;	0.012, 0.834	0.028 ± 0.009;	0.008, 0.049

CONTROL GROUP								
TIME POINTS (DAY)	IL-12P70		IL-1B		IL-2		IL-4	
	MEAN ± SE	CL (95%)	MEAN ± SE	CL (95%)	MEAN ± SE	CL (95%)	MEAN ± SE	CL (95%)
0	0.050 ± 0.017;	-0.003, 0.103	0.015 ± 0.015;	-0.033, 0.064	0.089 ± 0.042;	-0.044, 0.222	0.274 ± 0.232;	-0.464, 1.013
0.25	0.070 ± 0.020;	0.006, 0.135	0.012 ± 0.012;	-0.027, 0.052	0.030 ± 0.013;	-0.012, 0.072	0.258 ± 0.234;	-0.487, 1.002
1	0.078 ± 0.020;	0.014, 0.142	0.011 ± 0.011;	-0.023, 0.045	0.084 ± 0.045;	-0.059, 0.227	0.219 ± 0.199;	-0.414, 0.852
2	0.072 ± 0.020;	0.008, 0.137	0.009 ± 0.009;	-0.021, 0.040	0.018 ± 0.009;	-0.010, 0.047	0.260 ± 0.246;	-0.521, 1.042
3	0.078 ± 0.017;	0.023, 0.133	0.009 ± 0.009;	-0.021, 0.040	0.027 ± 0.016;	-0.022, 0.077	0.242 ± 0.228;	-0.484, 0.968
4	0.078 ± 0.026;	-0.005, 0.161	0.010 ± 0.010;	-0.023, 0.044	0.112 ± 0.059;	-0.076, 0.300	0.245 ± 0.226;	-0.473, 0.963
5	0.059 ± 0.017;	0.004, 0.115	0.010 ± 0.010;	-0.022, 0.042	0.113 ± 0.042;	-0.020, 0.246	0.223 ± 0.208;	-0.438, 0.884
7	0.080 ± 0.027;	-0.006, 0.167	0.006 ± 0.006;	-0.014, 0.026	0.146 ± 0.048;	-0.006, 0.299	0.223 ± 0.204;	-0.425, 0.871
14	0.075 ± 0.016;	0.024, 0.126	0.011 ± 0.011;	-0.024, 0.046	0.137 ± 0.013;	0.097, 0.177	0.247 ± 0.226;	-0.473, 0.967
21	0.043 ± 0.018;	0.018, 0.100	0.007 ± 0.007;	-0.015, 0.029	0.094 ± 0.023;	0.022, 0.167	0.193 ± 0.177;	-0.372, 0.757
SIV-INFECTED GROUP								
0	0.113 ± 0.041;	0.019, 0.206	0.048 ± 0.030;	-0.020, 0.116	0.364 ± 0.350;	-0.426, 1.155	0.067 ± 0.029;	0.001, 0.134
0.25	0.133 ± 0.037;	0.049, 0.217	0.008 ± 0.006;	-0.005, 0.022	0.242 ± 0.242;	-0.306, 0.790	0.069 ± 0.030;	0.001, 0.138
1	0.059 ± 0.024;	0.005, 0.112	0.001 ± 0.001;	-0.001, 0.003	0.017 ± 0.017;	-0.022, 0.057	0.320 ± 0.175;	-0.075, 0.715
2	0.082 ± 0.034;	0.005, 0.159	0.002 ± 0.002;	-0.003, 0.007	0.021 ± 0.021;	-0.027, 0.069	0.403 ± 0.250;	-0.162, 0.968
3	0.053 ± 0.022;	0.003, 0.104	0.000 ± 0.000;	0.000, 0.000	0.007 ± 0.007;	-0.009, 0.024	0.303 ± 0.152;	-0.040, 0.646
4	0.068 ± 0.032;	-0.004, 0.141	0.002 ± 0.002;	-0.002, 0.006	0.014 ± 0.014;	-0.018, 0.046	0.418 ± 0.203;	-0.040, 0.877
5	0.043 ± 0.020;	-0.003, 0.090	0.000 ± 0.000;	0.000, 0.000	0.000 ± 0.000;	0.000, 0.000	0.127 ± 0.078;	-0.051, 0.304
7	0.101 ± 0.021;	0.054, 0.148	0.002 ± 0.001;	0.000, 0.005	0.158 ± 0.120;	-0.113, 0.429	0.065 ± 0.022;	0.015, 0.115
14	0.111 ± 0.031;	0.040, 0.182	0.007 ± 0.004;	-0.002, 0.016	0.188 ± 0.111;	-0.062, 0.438	0.098 ± 0.028;	0.036, 0.161
21	0.092 ± 0.023;	0.039, 0.144	0.012 ± 0.008;	-0.006, 0.031	0.127 ± 0.064;	-0.017, 0.272	0.115 ± 0.049;	0.003, 0.227

CONTROL GROUP								
TIME POINTS (DAY)	IL-5		IL-6		IL-8		GRO-A	
	MEAN ± SE	CL (95%)	MEAN ± SE	CL (95%)	MEAN ± SE	CL (95%)	MEAN ± SE	CL (95%)
0	0.251 ± 0.163;	-0.266, 0.769	0.294 ± 0.146;	-0.169, 0.758	0.012 ± 0.005;	-0.003, 0.027	31.05 ± 6.936;	8.974, 53.12
0.25	0.200 ± 0.109;	-0.146, 0.546	4.657 ± 3.837;	-7.553, 16.87	0.014 ± 0.007;	-0.009, 0.038	28.11 ± 5.148;	11.72, 44.49
1	0.249 ± 0.101;	-0.072, 0.569	0.536 ± 0.120;	0.153, 0.919	0.013 ± 0.006;	-0.005, 0.031	19.61 ± 1.983;	13.30, 25.92
2	0.207 ± 0.092;	-0.086, 0.501	0.370 ± 0.112;	0.013, 0.727	0.005 ± 0.005;	-0.010, 0.020	17.83 ± 3.666;	6.612, 29.49
3	0.224 ± 0.094;	-0.077, 0.524	0.659 ± 0.302;	-0.304, 1.621	0.004 ± 0.004;	-0.009, 0.017	28.93 ± 8.833;	0.820, 57.04
4	0.239 ± 0.092;	-0.055, 0.534	0.511 ± 0.136;	0.077, 0.945	0.012 ± 0.006;	-0.008, 0.033	19.29 ± 4.481;	5.034, 33.55
5	0.250 ± 0.077;	0.006, 0.495	0.405 ± 0.146;	-0.060, 0.871	0.010 ± 0.008;	-0.016, 0.036	18.47 ± 2.393;	10.86, 26.09
7	0.218 ± 0.093;	-0.080, 0.515	0.284 ± 0.115;	-0.082, 0.651	0.000 ± 0.000;	0.000, 0.000	14.95 ± 3.428;	4.043, 25.86
14	0.269 ± 0.092;	-0.024, 0.561	0.413 ± 0.110;	0.064, 0.762	0.003 ± 0.002;	-0.003, 0.009	40.18 ± 15.38;	-8.773, 89.13
21	0.195 ± 0.691;	-0.025, 0.415	0.320 ± 0.134;	-0.106, 0.746	0.003 ± 0.002;	-0.004, 0.011	23.42 ± 6.372;	3.139, 43.69
SIV-INFECTED GROUP								
0	0.323 ± 0.096;	0.105, 0.541	0.048 ± 0.030;	-0.020, 0.116	0.073 ± 0.065;	-0.073, 0.220	102.0 ± 20.21;	56.32, 147.8
0.25	0.411 ± 0.131;	0.115, 0.706	0.008 ± 0.006;	-0.005, 0.022	0.061 ± 0.052;	-0.056, 0.178	68.04 ± 8.363;	49.12, 86.96
1	0.031 ± 0.022;	-0.018, 0.080	0.001 ± 0.001;	-0.001, 0.003	0.001 ± 0.001;	-0.001, 0.003	95.77 ± 16.69;	58.02, 133.5
2	0.029 ± 0.023;	-0.023, 0.081	0.002 ± 0.002;	-0.003, 0.007	0.002 ± 0.002;	-0.003, 0.008	169.8 ± 49.33;	58.17, 281.3
3	0.026 ± 0.017;	-0.013, 0.065	0.000 ± 0.000;	0.000, 0.000	0.000 ± 0.000;	0.000, 0.000	97.19 ± 23.76;	43.45, 150.9
4	0.030 ± 0.022;	-0.020, 0.081	0.002 ± 0.002;	-0.002, 0.006	0.002 ± 0.002;	-0.002, 0.006	120.4 ± 37.14;	36.37, 204.4
5	0.020 ± 0.012;	-0.008, 0.047	0.000 ± 0.000;	0.000, 0.000	0.000 ± 0.000;	0.000, 0.000	390.0 ± 157.3;	34.10, 745.9
7	0.882 ± 0.592;	-0.457, 0.220	0.002 ± 0.001;	0.000, 0.005	0.058 ± 0.031;	-0.013, 0.129	367.6 ± 112.8;	112.4, 622.8
14	0.283 ± 0.078;	0.107, 0.460	0.007 ± 0.004;	-0.002, 0.016	0.035 ± 0.011;	0.009, 0.060	258.1 ± 49.67;	145.8, 370.5
21	0.944 ± 0.196;	0.500, 1.388	0.012 ± 0.008;	-0.006, 0.031	0.249 ± 0.226;	-0.262, 0.759	229.7 ± 109.8;	-18.61, 478.0

CONTROL GROUP								
TIME POINTS (DAY)	I-309		MCP-3		MIP-5		TRAIL	
	MEAN ± SE	CL (95%)	MEAN ± SE	CL (95%)	MEAN ± SE	CL (95%)	MEAN ± SE	CL (95%)
0	0.386 ± 0.233;	-0.355, 1.128	0.654 ± 0.489;	-0.902, 2.210	0.922 ± 0.031;	0.824, 1.021	5.010 ± 0.678;	2.853, 7.166
0.25	0.211 ± 0.211;	-0.460, 0.881	0.632 ± 0.598;	-1.273, 2.536	0.942 ± 0.009;	0.912, 0.972	3.797 ± 0.464;	2.319, 5.275
1	0.291 ± 0.169;	-0.245, 0.828	0.544 ± 0.495;	-1.031, 2.118	0.951 ± 0.018;	0.892, 1.010	3.140 ± 0.759;	0.723, 5.556
2	0.226 ± 0.226;	-0.492, 0.943	0.638 ± 0.587;	-1.231, 2.506	0.935 ± 0.011;	0.898, 0.972	3.204 ± 0.353;	2.080, 4.328
3	0.116 ± 0.116;	-0.253, 0.484	0.600 ± 0.485;	-0.944, 2.145	0.947 ± 0.011;	0.911, 0.982	3.465 ± 0.556;	1.695, 5.235
4	0.134 ± 0.134;	-0.293, 0.562	0.576 ± 0.504;	-1.028, 2.180	0.930 ± 0.011;	0.895, 0.965	2.863 ± 0.537;	1.152, 4.573
5	0.054 ± 0.054;	-0.117, 0.225	0.610 ± 0.567;	-1.195, 2.416	0.942 ± 0.015;	0.895, 0.989	3.459 ± 0.618;	1.492, 5.426
7	0.000 ± 0.000;	0.000, 0.000	0.529 ± 0.521;	-1.142, 2.200	0.940 ± 0.008;	0.913, 0.966	3.748 ± 0.591;	1.866, 5.629
14	0.054 ± 0.054;	-0.117, 0.225	0.584 ± 0.584;	-1.274, 2.441	0.962 ± 0.016;	0.909, 1.014	3.633 ± 0.389;	2.396, 4.871
21	0.323 ± 0.323;	-0.706, 1.353	0.587 ± 0.530;	-0.706, 2.274	0.939 ± 0.021;	0.872, 1.006	3.728 ± 0.638;	1.697, 5.760
SIV-INFECTED GROUP								
0	0.838 ± 0.704;	-0.754, 2.430	0.583 ± 0.379;	-0.274, 1.440	1.122 ± 0.194;	0.682, 1.562	5.837 ± 0.590;	4.502, 7.173
0.25	0.797 ± 0.517;	-0.372, 1.967	0.568 ± 0.327;	-0.172, 1.309	1.067 ± 0.150;	0.727, 1.407	3.705 ± 0.560;	2.438, 4.971
1	0.968 ± 0.582;	-0.348, 2.283	0.470 ± 0.325;	-0.264, 1.205	1.086 ± 0.149;	0.749, 1.423	5.319 ± 0.780;	3.553, 7.085
2	0.871 ± 0.523;	-0.312, 2.053	0.432 ± 0.298;	-0.243, 1.107	1.055 ± 0.146;	0.725, 1.385	6.219 ± 0.780;	4.454, 7.984
3	1.040 ± 0.503;	-0.098, 2.177	0.431 ± 0.264;	0.165, 1.028	1.035 ± 0.110;	0.786, 1.283	5.347 ± 0.610;	3.968, 6.726
4	0.537 ± 0.291;	-0.121, 1.195	0.333 ± 0.184;	-0.083, 0.749	0.988 ± 0.069;	0.832, 1.114	7.148 ± 0.559;	5.885, 8.412
5	0.446 ± 0.332;	-0.306, 1.198	0.286 ± 0.161;	-0.078, 0.650	1.015 ± 0.067;	0.863, 1.167	8.017 ± 0.816;	6.170, 9.864
7	0.678 ± 0.389;	-0.203, 1.559	0.515 ± 0.191;	0.083, 0.947	1.007 ± 0.069;	0.852, 1.163	8.777 ± 0.974;	6.573, 10.98
14	0.608 ± 0.270;	-0.003, 1.219	0.412 ± 0.165;	0.039, 0.786	0.953 ± 0.026;	0.893, 1.012	6.459 ± 0.574;	5.162, 7.757
21	0.518 ± 0.124;	0.237, 0.798	0.245 ± 0.091;	0.038, 0.452	0.933 ± 0.020;	0.889, 1.978	6.581 ± 1.606;	2.948, 10.21

CONTROL GROUP		
TIME POINTS (DAY)	MIF	
	MEAN ± SE (10^4)	CL (95%,10^4)
0	4.3 ± 0.7;	2.0, 6.7
0.25	2.9 ± 0.3;	1.9, 3.9
1	2.3 ± 0.2;	1.7, 2.9
2	2.8 ± 0.4;	1.4, 4.1
3	2.3 ± 0.3;	1.4, 3.3
4	2.2 ± 0.2;	1.7, 2.7
5	3.7 ± 0.6;	1.6, 5.7
7	2.3 ± 0.04;	2.2, 2.5
14	3.7 ± 1.2;	-0.07, 7.5
21	2.5 ± 0.6;	0.5, 4.5
SIV-INFECTED GROUP		
0	11.6 ± 3.5;	3.6, 19.6
0.25	5.6 ± 0.5;	4.4, 6.8
1	5.2 ± 0.5;	4.1, 6.4
2	7.8 ± 2.3;	2.6, 13.1
3	7.1 ± 2.5;	1.4, 12.8
4	7.0 ± 1.8;	2.9, 11.2
5	9.6 ± 5.0;	-1.8, 20.9
7	11.4 ± 4.0;	2.4, 20.5
14	6.7 ± 1.0;	4.5, 9.0
21	6.6 ± 1.0;	4.4, 8.8
