# Supplementary figures and images for: Divergent Cytokine and Chemokine Responses at Early Acute Simian Immunodeficiency Virus Infection Correlated with Virus Replication and CD4 T Cell Loss in a Rhesus Macaque Model
Source: Vaccines (Basel). 2023 Jan 25;11(2):264. doi: 10.3390/vaccines11020264 (PMC9963901; doi:10.3390/vaccines11020264)

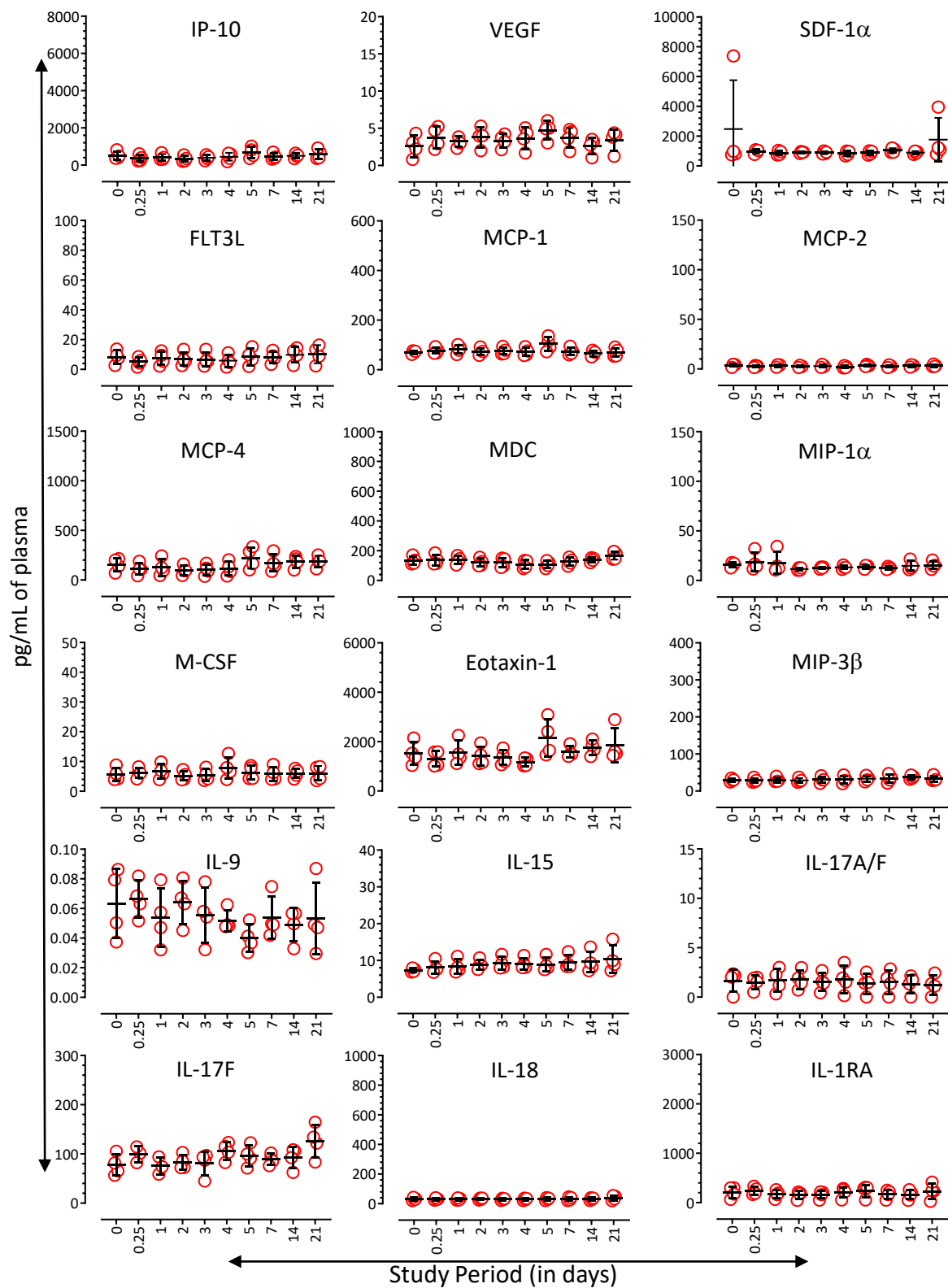

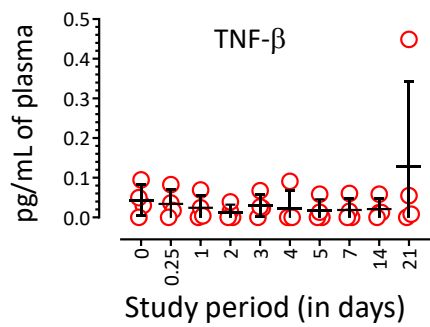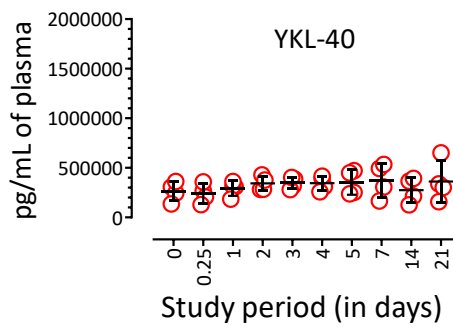

Supplement: Supplementary file 1 [file vaccines-11-00264-s001.zip › Supplementary Figure S1.pdf]
